# Recycling of Poly(lactic acid): From Molecular Degradation to Circular End-of-Life Strategies

**DOI:** 10.3390/polym18141731

**Published:** 2026-07-15

**Authors:** Hasan Saygin, Asli Baysal

**Affiliations:** 1 Application and Research Center for Advanced Studies, Istanbul Aydin University, Sefakoy Kucukcekmece, 34295 Istanbul, Türkiye; 2 Department of Chemistry, Faculty of Science and Letters, Istanbul Technical University, Maslak, 34469 Istanbul, Türkiye; asli.baysal@itu.edu.tr

**Keywords:** poly(lactic acid), mechanical recycling, post-consumer bioplastics, life-cycle assessment, product safety, circular economy

## Abstract

Poly(lactic acid) (PLA) is widely recognized as a biodegradable bioplastic, yet reliance on industrial composting alone can forfeit embedded material and energy value when recovery is technically feasible. Recycling can retain this value, but PLA performance is affected by service-life aging, hydrolytic cleavage, thermal and shear history, and contamination. Whereas previous literature often treats end-of-life routes separately, this review integrates mechanical reprocessing, reactive upgrading, chemical and hydrothermal depolymerization, and life-cycle assessment within a feedstock–process–structure–performance–safety–circularity framework. We examine how molar mass, rheology, crystallinity, and mechanical performance evolve during recycling, and compare upgrading strategies, including chain extenders, plasticizers, blends, and fillers, in terms of property restoration, recyclability, migration, and ecotoxicity trade-offs. Chemical and hydrothermal routes are evaluated according to monomer yield, stereochemical purity, additive tolerance, repolymerization potential, and process severity. Life-cycle evidence shows that circularity cannot be defined solely by climate impact or biodegradability, as burden shifting may occur in terms of toxicity, energy demand, land use, and resource consumption. Accordingly, we propose a decision map linking feedstock quality with suitable routes and target applications. Overall, clean, dry, and traceable PLA should be prioritized for mechanical recycling, whereas degraded or contaminated streams require evidence-based upgrading or depolymerization instead of default disposal or composting practices.

## 1. Introduction

Poly(lactic acid) (PLA) is a widely used bio-based and biodegradable/industrially compostable polyester, with growing relevance in discussions of fossil-resource use, plastic pollution, and climate change. PLA is produced from renewable agricultural feedstocks such as corn, sugarcane, and sugar beet through the fermentation of carbohydrates to lactic acid, followed by oligomerization and ring-opening polymerization of lactide to obtain high-molecular-weight polymer chains [1,2,3]. This production route can partly decouple polymer manufacturing from petroleum resources and may reduce selected climate and fossil-resource impacts depending on feedstock, energy mix, and system boundary. As an aliphatic polyester thermoplastic, PLA can be processed using established industrial techniques, including extrusion, injection molding, thermoforming, and fused filament fabrication (FFF), which has supported its commercial adoption.

Due to its balance of renewability, processability, and functional properties, PLA is utilized across various sectors. In packaging, PLA is used in films, trays, cups, and disposable food containers, benefiting from its transparency, stiffness, and suitability for selected food-contact applications [1,2]. In biomedical and pharmaceutical applications, PLA’s biocompatibility and controlled biodegradability enable its use in sutures, orthopedic implants, tissue-engineering scaffolds, and controlled drug-delivery systems [2,4,5]. Additionally, PLA is widely used in desktop FFF and selected industrial 3D-printing applications because of its relatively low processing temperature, dimensional stability, and ease of use, supporting applications ranging from rapid prototyping to functional consumer products [6,7,8]. These applications highlight PLA’s versatility but also generate increasingly complex and heterogeneous waste streams.

Despite being widely labeled as biodegradable, the growth in PLA consumption raises important concerns regarding end-of-life management. Industrial composting, often presented as a disposal route, requires controlled temperature, moisture, and microbial conditions that are not universally available in municipal waste-management systems [9,10]. Under ambient environmental conditions or in landfills, PLA degradation can be slow, creating a risk of persistence and accumulation [11]. Furthermore, improper disposal of PLA into traditional plastic recycling streams can negatively affect the quality of recycled polyolefins and polyethylene terephthalate, complicating sorting and processing operations [12,13]. Together, these limitations indicate that biodegradability alone does not guarantee environmental sustainability and that recycling strategies are important for responsible PLA management.

Recycling has therefore become an important component in advancing a circular economy for PLA. Several end-of-life routes have been explored, including mechanical recycling, chemical recycling, biological or enzymatic recycling, and composting. Mechanical recycling is often a comparatively accessible option when clean PLA streams, sorting capacity, and suitable processing infrastructure are available; it involves the collection, sorting, grinding, and remelting of PLA waste. However, repeated thermal and mechanical processing can lead to chain scission, molecular-weight reduction, and the deterioration of mechanical and thermal properties, limiting the number of viable recycling cycles [14,15]. Chemical recycling offers an alternative by depolymerizing PLA into monomers or value-added intermediates via hydrolysis, alcoholysis, or solvent-based processes, and can enable the recovery of raw materials for repolymerization when purity and stereochemical requirements are met [16,17]. While promising for contaminated or mixed PLA waste, these processes often require higher energy input and careful management of solvents and catalysts. More recently, biological and enzymatic recycling approaches have gained attention as potentially selective, lower-temperature pathways for PLA depolymerization, although challenges related to reaction rates, enzyme stability, and scalability remain [18,19].

Two additional factors strengthen the rationale for recycling and reuse. First, PLA is now processed not only by extrusion and injection molding, but also by fused-filament fabrication; therefore, pre-consumer scrap, failed prints, support structures, and off-spec parts may undergo repeated heating, shear, and drying histories before becoming waste. Second, PLA production depends on carbohydrate feedstocks and purification-intensive lactic acid/lactide routes. High-molecular-weight PLA requires high monomer purity, while first-generation plant raw materials, regional sugar availability, scale effects, and final resin price can restrict broader deployment [20]. Under these conditions, recycling can extend the service life of invested biomass and energy processing, and chemical recycling can return PLA waste to lactic acid, lactide, or alkyl-lactate intermediates when purity and stereochemistry are controlled [21].

Although numerous studies and reviews have addressed individual aspects of PLA production, properties, and biodegradation, comparative evaluations of PLA recycling routes remain dispersed across the literature [1,9,11]. Many existing works focus on single end-of-life options, such as composting or mechanical recycling, without consistently comparing their technical performance, environmental trade-offs, and suitability for different PLA grades and application-derived waste streams [10,22]. Moreover, the growing diversity of PLA applications, particularly in additive manufacturing and biomedical fields, has introduced waste streams with distinct processing histories and additive compositions that are less consistently addressed in earlier assessments.

This review contributes an integrated and application-driven evaluation of PLA recycling methods, encompassing mechanical, chemical, biological/enzymatic recycling, and composting within a unified framework. Rather than treating these approaches in isolation, this work examines how processing history, additive content, thermal degradation, and waste quality influence recyclability and material performance [12,14,15]. Particular emphasis is placed on recent advances in chemical recycling and biorecycling strategies that enable monomer recovery or selective depolymerization, highlighting their possible role in closing material loops for lower-grade or contaminated PLA waste [16,19,23].

Beyond technological comparison, the scope of this review incorporates environmental performance, economic feasibility, and infrastructure constraints associated with each recycling pathway. Life-cycle assessment outcomes, scalability considerations, and compatibility with existing waste-management systems are discussed to provide a realistic appraisal of implementation potential [16,22,24]. By identifying knowledge gaps and research priorities, this review aims to provide structured considerations for advancing PLA recycling and supporting the transition of PLA from a predominantly single-use bioplastic toward a more circular material system.

**Figure 1:**
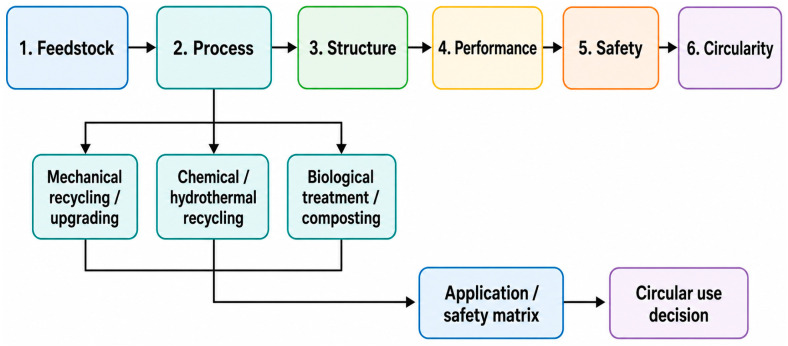
Integrated framework for PLA recycling and circularity assessment.

To make the logic of the review explicit, Figure 1 presents the integrated feedstock–process–structure–performance–safety–circularity framework used to organize the evidence. The framework begins with the diversity of PLA-containing feedstocks, including neat post-industrial material, post-consumer products, blended or contaminated streams, and filled or additivated formulations. These inputs determine the feasible processing route, including sorting and pretreatment, mechanical reprocessing, upgrading/compounding, chemical or hydrothermal depolymerization, and biological treatment. Each route modifies structure-sensitive descriptors such as molecular weight, dispersity, crystallinity, stereochemical integrity, thermal transitions, morphology, and contaminant burden. These structural changes then determine processability and performance, including melt flow behavior, viscosity, tensile and impact properties, barrier performance, and thermal stability. Safety and circularity are treated as cross-cutting filters because food-contact suitability, additive migration, ecotoxicity, recyclate substitution, and burden shifting ultimately determine whether a recycled PLA stream can be used in high-value applications or should be redirected to lower-demand products or alternative end-of-life routes.

The studies compiled in this review show that PLA circularity is governed by a narrow balance between molecular degradation, processability, performance retention, and end-of-life trade-offs. Repeated melt reprocessing can rapidly reduce molecular integrity: for example, PLA 2003D lost approximately 78% of its molecular weight over five extrusion cycles, accompanied by an increase in melt flow index from 10.60 to 18.20 g/10 min [1]. Similarly, Ingeo 3025D showed a severe rise in melt flow rate from approximately 18 to 277 g/10 min by the seventh cycle, after which further cycles became difficult to measure [2]. Bottle-grade PLA also showed substantial degradation during repeated recycling, with molecular weight decreasing by approximately 40% after six cycles while crystallinity increased from 6.9% to 39.5% [3]. These findings indicate that mechanical recycling is feasible but strongly degradation-limited, especially when moisture control, thermal history, and residence time are not optimized.

Upgrading strategies can partially compensate for this degradation. For instance, 2 wt% halloysite nanotube addition increased the tensile strength of recycled PLA from 42.98 to 49.39 MPa, while 5 wt% increased the degradation temperature from 452.12 to 465.58 °C [4]. However, such improvements should not be interpreted only as property recovery, because additives, fillers, and compatibilizers may also affect food-contact suitability, sorting behavior, and subsequent recyclability.

Chemical recycling provides an alternative route for recovering molecular value when mechanical recycling reaches its practical ceiling. Representative organocatalytic systems show high conversion, oligomer or monomer recovery, catalyst reuse, and in some cases scale-up potential. Diphenyl phosphate catalysis, for example, produced oligomers with DP <3, achieved >85% conversion, allowed ten catalyst-reuse cycles, and was verified at kg scale [5]. The p-BNPP route further illustrates the importance of stereochemical retention and waste tolerance, reaching efficient PLA conversion within 30 min at 160 °C while retaining the L-configuration and tolerating durable-plastic contaminants [6].

Environmental assessment confirms that circularity cannot be judged by climate change alone. Prospective LCA results show that mechanical recycling can provide the lowest climate-change impact, ranging from −1.22 to −0.67 kg CO_2_-eq/kg PLA, whereas chemical recycling without repolymerization can reach up to +0.58 kg CO_2_-eq/kg PLA [7]. At the same time, burden shifting remains important: composting increased terrestrial ecotoxicity by 31%, and recycling pathways increased water consumption by up to 49% under some scenarios [7]. Therefore, the quantitative evidence supports a tiered decision logic: prioritize high-quality mechanical recycling when degradation and contamination are controlled; apply upgrading only when it preserves safety and future recyclability; use chemical recycling for degraded or mixed streams where stereochemical quality can be retained; and evaluate composting or biological treatment only for application-specific cases where material recovery is not realistic.

## 2. Materials and Methods

**Figure 2:**
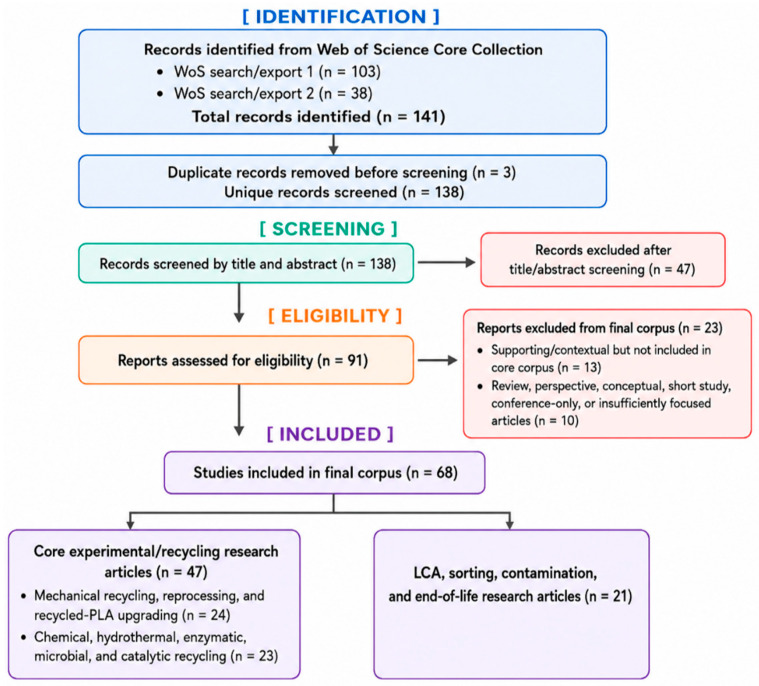
PRISMA 2020 [25] flow diagram for selecting research articles on PLA recycling, upgrading, chemical recovery, sorting, contamination, and end-of-life assessment.

We conducted a structured literature search informed by the Preferred Reporting Items for Systematic Reviews and Meta-Analyses (PRISMA) guidelines [25]. The literature search was conducted in two stages: an initial search in February 2026 and an update search in May 2026; Web of Science (WoS) Core Collection was used as the sole database because it provides a curated, multidisciplinary, and reproducible source suitable for transparent PRISMA-based screening. Figure 2 presents the flowchart of this study. The following keywords were used: “recycling”, “polylactic acid”, and “PLA”. Eligible studies addressed PLA recycling, sorting, reprocessing, degradation/depolymerization, or life-cycle/end-of-life assessment with sufficient methodological detail. Records were excluded if they were duplicates, not PLA-focused, outside the recycling/end-of-life scope, review/editorial/conference-only items, or lacked usable technical data. To focus the search on PLA recycling, English-language peer-reviewed research articles were included in the core corpus, while gray literature, seminars, non-English publications, editorials, and reports from government and non-government organizations were also excluded from the core corpus. For the core review corpus, WoS records were screened using a two-stage selection process. First, duplicate records were removed from the combined WoS exports. Titles and abstracts were then evaluated for relevance to PLA recycling, recycled PLA upgrading, chemical or biological depolymerization, sorting, contamination, life-cycle assessment, or end-of-life management. Studies were retained in the two search corpuses when they provided original experimental, process, sorting, contamination, safety, modeling, or LCA data directly relevant to PLA or PLA-containing bioplastic recycling. General reviews, conceptual papers, conference-only records, application papers without recycling or end-of-life evidence, and studies where PLA-specific findings could not be separated were excluded from the final core corpus, although selected contextual papers may still be used in the introduction or discussion. The final corpus contained 68 studies: 47 core recycling or reprocessing research articles and 21 LCA, sorting, contamination, or end-of-life research articles.

## 3. PLA Characteristics Governing Recyclability

The recyclability of PLA is influenced by a combination of molecular, thermal, morphological, and compositional characteristics, rather than solely by its bio-based origin. Although PLA is a thermoplastic polyester and can therefore be mechanically reprocessed, its ester backbone is sensitive to hydrolytic, thermal, and thermomechanical scission during service life, washing, drying, extrusion, injection molding, and repeated reprocessing. Four important processing-related variables are residual moisture, melt temperature, residence time, and shear; together, these influence the extent of molecular-weight loss and the resulting decline in melt strength, viscosity, and end-use performance [26].

Molecular weight is a major determinant of mechanical recyclability. During recycling, PLA chains are cleaved into shorter fragments, leading to reductions in number- and weight-average molecular weight, intrinsic viscosity, complex viscosity, and zero-shear viscosity, while melt flow index generally increases [26,27]. This behavior is important because most industrial processing operations require a sufficiently narrow viscosity window. A moderate reduction in molecular weight can improve flowability, but excessive degradation produces brittle recycled PLA with reduced tensile strength, lower thermal stability, weaker melt strength, and poorer suitability for extrusion, thermoforming, injection molding, or 3D-printing applications. Therefore, molecular-weight retention, usually monitored by GPC, intrinsic viscosity, MFI, or rheology, is a useful indicator of whether a PLA stream can be recycled directly or would benefit from upgrading by chain extension, branching, blending, or solid-state polymerization [26,28].

Moisture sensitivity is another important limitation. PLA hydrolysis occurs through the cleavage of ester bonds, producing shorter oligomers and increasing carboxyl end-group concentration. These acidic end groups can further catalyze hydrolysis, making PLA degradation partly autocatalytic. Hydrolytic cleavage often starts in amorphous regions, which can cause an apparent increase in crystallinity as the more accessible amorphous fraction is degraded first [27]. Consequently, post-consumer PLA exposed to humid storage, wet food residues, alkaline washing, or incomplete drying may show reduced molecular weight even before melting reprocessing. Drying before extrusion is therefore not only a processing step but also a recyclability requirement; insufficient drying may increase apparent fluidity, but this reflects degradation rather than improved material quality [27].

Crystallinity and stereochemistry also influence degradation and reuse options. Commercial PLA grades differ in L-/D-lactide content, optical purity, crystallization rate, melting behavior, and heat resistance. More stereoregular PLA grades, such as high-L-content PLLA, crystallize more readily and generally exhibit higher thermal resistance, whereas PLA grades with higher D-lactide content tend to remain more amorphous and transparent but may have lower heat resistance. During recycling, chain scission can increase chain mobility and promote crystallization, which may increase stiffness or hardness while simultaneously reducing toughness and tensile performance. This means that crystallinity can partially mask molecular degradation if only mechanical stiffness is considered. For this reason, recyclability assessments should combine molecular-weight or viscosity data with DSC, Fourier-transform infrared (FTIR) crystallinity markers, tensile properties, and thermal stability rather than relying on a single endpoint [26,28].

Spectroscopic techniques, specifically FTIR and Raman spectroscopy, provide robust, non-destructive methodologies for monitoring the hydrolytic and thermomechanical chain scission of PLA during multiple reprocessing cycles [26]. While macroscopic molecular-weight reduction is typically quantified by size-exclusion chromatography, vibrational techniques provide molecular-level fingerprints of degradation mechanisms. Raman spectroscopy is sensitive to changes in the ester backbone and crystalline/amorphous organization, whereas FTIR spectroscopy can capture the accumulation of hydroxyl and carboxyl end groups associated with chain scission and subtle structural shifts between crystalline and amorphous phases. Combined with GPC, DSC, rheology, and mechanical testing, these methods help distinguish true property recovery from stiffness changes that merely reflect crystallization or additive effects.

Thermal stability helps define the practical recycling window. PLA typically shows a glass transition around 60 °C and melting behavior in the approximate range used for melt processing; however, degradation accelerates as processing temperature, residence time, and shear increase. Intramolecular transesterification, backbiting, random chain scission, hydrolysis, and thermomechanical degradation can occur during melt processing, especially at high temperatures or in the presence of residual moisture, oxygen, catalysts, pigments, or metal-containing impurities [26,27]. Thus, repeated extrusion at excessive temperature or long residence time progressively lowers molecular weight and alters crystallization behavior. From a recycling perspective, lower effective melt temperatures, short residence times, controlled shear, efficient drying, and minimized oxygen/moisture exposure are important for maintaining recycled PLA quality [26].

Waste-stream purity and formulation history are also important. PLA waste from a known single grade is generally easier to recycle than mixed post-consumer PLA because pigments, plasticizers, nucleating agents, mineral fillers, fibers, residual catalysts, food residues, labels, and other polymers introduce heterogeneous degradation and crystallization behavior. Studies on 3D-printing PLA waste show that mechanical recycling can decrease intrinsic viscosity and increase crystallinity, while mixed PLA waste streams may show greater variability than well-defined reference grades [28]. Additives can be beneficial during the first product life, but they complicate subsequent recycling if they change crystallization, promote chain scission, alter color, lower thermal stability, or interfere with chain-extension reactions. Therefore, design-for-recycling of PLA should prioritize identifiable PLA grades, limited pigment complexity, known additive packages, and separation from PBAT, PBS, PHB, PET, polyolefins, and multilayer materials unless compatibilization is specifically planned [28].

Chemical recyclability is enabled by the ester backbone, but product quality depends on stereochemical and feedstock control. PLA can be depolymerized to lactic acid, lactate esters, lactide, or other value-added products depending on the catalyst, solvent, temperature, and reaction medium. Among these options, direct recovery of lactide can be attractive because lactide can be repolymerized to PLA of suitable quality when purity and stereochemical requirements are met, offering a more closed-loop route than downcycling or composting [29]. However, chemical recycling is sensitive to racemization, residual additives, moisture, contaminants, catalyst residues, and mixed-polymer feedstocks. Recovery of optically pure L-lactide is particularly important when the goal is repolymerization to high-performance PLLA. Recent studies also show that post-consumer PLA can be converted into valuable platform chemicals such as lactide and acrylic acid using selected catalytic systems, while methanolysis routes can produce methyl lactate that can be further converted back toward PLA precursors [30,31].

Overall, PLA recyclability is controlled by the balance between molecular-weight retention, moisture history, thermal-processing severity, crystallinity, stereochemical purity, additive content, and waste-stream homogeneity. Mechanical recycling is generally most feasible when PLA is clean, dry, compositionally known, and processed under mild conditions. When molecular weight has already declined, upgrading strategies such as chain extension, branching, solid-state polymerization, or controlled blending may be required [1,28]. For highly degraded, contaminated, or mixed PLA streams, chemical recycling can provide a more suitable circular route, provided that stereochemical integrity and product purification can be maintained.

## 4. Mechanical Recycling of Neat and Post-Consumer PLA

**Figure 3:**
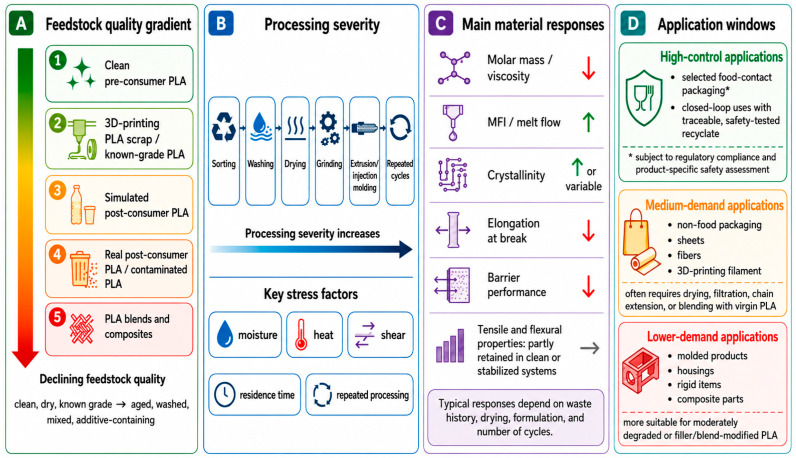
Mechanical recycling map for PLA showing how feedstock quality and processing severity control molecular degradation, melt flow behavior, crystallinity, mechanical retention, barrier performance, and potential application windows. Detailed study-level evidence is provided in Supplementary Table S1.

Detailed study-level data on the mechanical recycling of neat, pre-consumer, simulated post-consumer, and blend/composite PLA are provided in Supplementary Table S1. To improve readability, Figure 3 summarizes the main evidence as a feedstock quality–processing severity–property retention map. The figure highlights that clean, dry, and compositionally known PLA streams generally offer the most reliable mechanical recycling window, whereas aged, washed, contaminated, or repeatedly processed PLA streams more often require stabilization, chain extension, blending, or lower-demand applications [32,33,34,35,36,37,38,39,40,41,42,43,44,45,46,47,48,49,50,51,52,53,54].

A useful distinction can be made between primary/pre-consumer recycling and secondary/post-consumer recycling. For example, Munoz-Shuguli et al. [32] investigated how a commercial PLA water bottle preforms when subjected to six consecutive extrusion cycles. They reported progressive degradation, with molecular weight decreasing by up to 40%, crystallinity increasing from 6.9% to 39.5%, and barrier properties being substantially affected after repeated processing. Similarly, Nesic et al. [33] studied additive-free pre-consumer PLA bottle waste over 10 processing cycles and found that MFI increased from approximately 8.7 to 13.9 g/10 min, while tensile strength remained relatively stable, from about 68.2 MPa after the first cycle to 67.1 MPa after the tenth cycle. These results suggest that clean pre-consumer PLA can tolerate repeated processing better than aged or contaminated post-consumer streams under controlled conditions.

Earlier multiple-processing studies remain important because they established repeated extrusion and injection molding as controlled models of recycling-induced aging rather than simple remelting. These works support the use of molecular-weight loss, intrinsic-viscosity decline, MFI/MFR increase, color change, and embrittlement as core indicators of PLA reprocessing damage. Recent studies on FFF/3D-printing waste extend the same logic to failed prints and filament scrap, where prior drying, residence time in the nozzle, pigment packages, and repeated melt histories may strongly affect recyclate quality [14,15,26,27,28].

In contrast, secondary or simulated post-consumer PLA generally showed more pronounced degradation. Arjona et al. [34] simulated post-consumer recycling of commercial PLA water bottles using UVB exposure for 40 h, thermal aging at 50 °C for 468 h, hydrothermal aging at 25 °C for 240 h, alkaline/surfactant washing at 85 °C, and extrusion at 180 °C. Although tensile strength and modulus were largely retained, MFI increased from 14.54 to 15.53 g/10 min, elongation at break decreased, and oxygen and water-vapor permeability increased. This indicates that service-life aging and washing can compromise barrier performance even when mechanical properties remain acceptable. Morán et al. [35] further showed that post-consumer rPLA could be upgraded with additives: OLA-based chain extender retained tensile strength around 64 MPa and limited migration risk, whereas high plasticizer loading improved flexibility but reduced stiffness and increased migration concerns.

Repeated processing of neat PLA usually resulted in increased melt flow or reduced viscosity, consistent with chain scission. Shojaeiarani et al. [36] reported that PLA 2003D lost about 78% of its molecular weight after five extrusion cycles, decreasing from 203,500 to 44,149 g/mol, while MFI increased from 10.60 to 18.20 g/10 min. Nomadolo et al. [37] similarly observed that PLA LX175 could not be processed beyond the sixth cycle because of low melt strength; tensile strength decreased by about 10% by the sixth cycle, and strain at break decreased by about 44% already by the second cycle. Nemeth et al. [38] reported a stronger MFR rise for injection-molded Ingeo 3025D PLA, from about 18 g/10 min after the first cycle to 277 g/10 min after the seventh cycle, after which MFR could no longer be measured under the applied conditions. These examples support the use of melt flow change as a sensitive indicator of PLA degradation during mechanical recycling.

Several studies also suggest that blending can improve the practical recyclability of PLA-based materials. Farias et al. [39] examined PLA/PHB 70/30 blends recycled up to five times and reported significant viscosity reduction but only a limited tensile strength decrease of about 5%. The retention of tensile properties was attributed to increased crystallinity and finer PHB-domain morphology during recycling. Plavec et al. [40] also reported that a PLA/PHB 45/55 blend could be extruded repeatedly up to 11 cycles, with complex viscosity decreasing but tensile strength remaining broadly stable in the range of about 23–35 MPa. In PLA/PBAT systems, Nemeth et al. [38] showed that mixed PLA/PBAT industrial waste could be recycled, but the final rheological, thermal, and mechanical properties depended strongly on the PLA/PBAT ratio. These studies suggest that blend morphology and phase composition can partly compensate for PLA chain scission.

Additive-based upgrading is another important route. Beltrán et al. [41] used chain extender and dicumyl peroxide in simulated recycled PLA and showed that additive efficiency depended on the initial degradation state of the material. Barletta et al. [42] demonstrated that a Joncryl-type chain extender could reduce MFI and improve elongation at break in PLA-based post-process material intended for cast extrusion and thermoforming. Benvenuta-Tapia and Vivaldo-Lima [43] used RAFT-derived epoxy-functional chain extenders and reported recovery of molar mass and complex viscosity in recycled PLA. These examples support the use of reactive extrusion as a targeted upgrading strategy when mechanical recycling alone produces excessive melt-strength loss.

Barrier properties were less frequently reported than molecular, rheological, or mechanical properties. Where measured, however, barrier deterioration was reported in bottle-grade PLA. Arjona et al. [34] found that oxygen permeability increased after aging, recycling, and reprocessing, while Munoz-Shuguli et al. [32] reported that both oxygen and water-vapor barrier properties were substantially affected by repeated primary reprocessing. Therefore, although recycled PLA can retain acceptable tensile properties in some cases, high-barrier applications such as bottle-to-bottle recycling may require additional stabilization, chain extension, blending, or coating strategies.

Overall, the literature examples in Table S1 indicate that PLA mechanical recycling is generally more reliable for clean, dry, and homogeneous pre-consumer waste. Post-consumer PLA is more challenging because aging, washing, hydrolysis, and repeated extrusion accelerate molecular degradation and barrier loss. Blends such as PLA/PHB, PLA/PBAT, PLA/PBS, PLA/aPHA, and PLA/PCL, as well as reactive chain extenders, peroxides, and fillers, can improve selected properties, but their effectiveness depends on the degradation level of the recycled PLA and the final application requirements.

## 5. Upgrading Strategies for Recycled PLA

Table 1 summarizes the main strategies reported for upgrading recycled PLA and PLA-rich recyclates [32,33,34,35,36,37,38,39,40,41,42,43,44,45,46,47,48,49,50,51,52,53,54]. The simplest reference condition is additive-free reprocessing, which is useful for evaluating the intrinsic recyclability of clean pre-consumer or commercial PLA streams. However, repeated melt processing generally promotes chain scission, lower viscosity or molecular weight, higher melt flow, crystallinity changes, and color deterioration, especially after several cycles [32,33,36,38]. Therefore, upgrading is often required when recycled PLA is intended for applications beyond low-demand reuse.

Reactive upgrading should be treated as a chemistry-specific process-control step. Epoxy-functional chain extenders can react with both carboxyl and hydroxyl end groups and may generate secondary hydroxyl groups with lower subsequent reactivity; therefore, acid number, hydroxyl number, residual moisture, residence time, and additive dose should be controlled during reactive extrusion. Isocyanate and oxazoline chain extenders are less frequently emphasized in recycling-focused studies but are relevant alternatives: isocyanates can form polyester–urethane structures, whereas oxazolines can introduce amide-containing polyester structures. These reactions may increase molar mass or toughness, but they also require assessment of residual reactants, worker-safety handling, migration potential, chemical-recycling compatibility, and future recyclability [55].

Reactive modification is one of the more frequently reported approaches for restoring the processing window of degraded PLA. Epoxy-functional and multifunctional chain extenders react with hydroxyl and carboxyl end groups formed during PLA degradation, thereby increasing molar mass, melt viscosity, and melt strength [35,41,42,43,49,56]. These additives are particularly relevant for extrusion, sheet production, thermoforming scrap recovery, and post-consumer packaging recyclates. Organic peroxides can also improve viscosity and thermal resistance through radical-mediated branching or crosslinking, but their effect is more difficult to control because chain extension and further degradation may occur simultaneously [41]. Solid-state polymerization is another potential route for rebuilding molecular weight while preserving the PLA chemical identity, although it requires clean, well-dried, and well-sorted material and is slower than melt-based upgrading [32,56].

Nonreactive or formulation-based strategies mainly aim to compensate for brittleness or improve application-specific properties. Plasticizers increase flexibility, chain mobility, and film-forming ability, but their use introduces possible safety and recyclability trade-offs because migration, volatility, and property drift can occur during storage or later recycling cycles [35,40,54]. Compatibilizers are especially important in PLA/fiber and PLA/polymer blends because they improve interfacial adhesion and phase dispersion; however, they also increase chemical complexity and may affect sorting, biodegradation, and future closed-loop recycling [49,50,51,52,53]. Fiber and mineral fillers, including natural fibers, halloysite nanotubes, silk fibroin, chitosan, talc, and TiO_2_, can increase stiffness, strength, crystallinity, and thermal resistance, but excessive loading or poor dispersion may reduce elongation and complicate repeated recycling [44,47,51,52,53].

Blending is another common upgrading route. PLA blends with PBAT, PHB, PBS, PCL, or aPHA can improve toughness, flexibility, crystallization behavior, or barrier performance, depending on blend ratio and morphology [37,38,39,40,48]. Several studies show that PLA/PHB and PLA/PBAT-rich materials can tolerate repeated recycling under controlled ratios and processing conditions, whereas immiscibility, viscosity loss, and phase separation remain important limitations [37,38,39,40,48]. Blending recycled PLA with virgin PLA is a practical strategy for controlled industrial scrap because it dilutes degradation, but blending with conventional polymers such as HDPE or PC can compromise PLA purity, compostability, and closed-loop recyclability [34,46,50]. Overall, Table 1 indicates that no single upgrading method is universally optimal. Chain extenders provide a direct route for restoring melt properties, plasticizers are useful for flexibility but raise migration concerns, fillers and fibers provide reinforcement but complicate sorting, and biodegradable polyester blends offer property balancing when morphology and composition are carefully controlled.

Several representative studies illustrate how the upgrading route determines both the performance gain and the recycling trade-off of rPLA. Beltran et al. [41] provide a representative example of reactive upgrading using a chain extender and dicumyl peroxide (DCP). In their study, PLA was first converted into simulated post-consumer material through photochemical, thermal, hydrothermal, and washing steps, while a second batch was severely hydrolyzed to represent highly degraded waste. Both residues were then melt reprocessed with different amounts of a PLA-based chain extender or DCP. The additives reacted with degraded PLA end groups and promoted chain extension, branching, and crosslinking, which improved viscosity, thermal stability, and microhardness. However, the same study also showed that the effect was strongly dependent on additive dosage and the previous degradation state of the PLA; higher peroxide loading could further reduce thermal stability because radical reactions also promoted chain scission. This example is therefore useful for the table because it shows both the benefit and the trade-off of reactive additives: they can restore useful properties, but only within a controlled processing window.

A second representative example is the post-consumer packaging study of Moran et al. [35], because it combines functional upgrading with safety evaluation. Commercial PLA water bottles were subjected to accelerated aging and recycling simulation, followed by melt extrusion with a Glyplast OLA 2 plasticizer at 20 and 30 wt% or Glyplast OLA 550 chain extender at 2 and 5 wt%. The plasticizer increased flexibility and chain mobility, with the highest plasticizer content producing a substantial ductility improvement; however, it also caused stronger molecular-weight reduction and raised migration concerns. In contrast, the chain extender formulations did not provide the same plasticizing effect, but they gave a more favorable safety profile, with overall migration values below the established limit and lower cytotoxicity concern than the high plasticizer formulation. This study is useful for Table 2 because it demonstrates why property improvement alone is not sufficient for recycled PLA intended for food packaging: migration and cell-based toxicity data are important when additives are used.

For filler-based upgrading, Gnanasambandam et al. [47] showed that halloysite nanotubes can improve selected mechanical and thermal properties of recycled PLA composites. The authors used rPLA obtained from failed or unused 3D-printing objects and leftover filament spools, then compounded it with 1–5 wt% unmodified halloysite nanotubes. The most balanced mechanical improvement occurred at moderate filler loadings: tensile strength increased from 42.98 MPa for neat rPLA to 49.39 MPa at 2 wt% HNT, flexural strength peaked at 78.54 MPa at 3 wt% HNT, and tensile modulus increased progressively up to 2971.26 MPa at 5 wt% HNT. Thermal resistance also improved, with degradation temperature increasing from 452.12 °C for rPLA to 465.58 °C at 5 wt% HNT, while char residue rose from 4.23% to 9.96%. The trade-off was that higher filler contents could cause agglomeration, which limited tensile strength despite the thermal barrier effect. This example supports the table entry that mineral fillers can improve stiffness, strength, crystallinity, and thermal stability, but only if dispersion is controlled.

Blending with biodegradable polyesters is illustrated by Farias et al. [39], who studied PLA/PHB 70/30 wt% blends over five mechanical recycling cycles. Although melt flow increased and viscosity decreased with repeated extrusion, FTIR did not show major spectral evidence of chain scission, and the morphology became more homogeneous. The authors attributed this to limited interfacial changes and finer PHB droplets, which acted as nucleating domains. As a result, PLA crystallinity increased from about 6% in the control to 17.5% after five recycling cycles, while the reduction in tensile strength was not significant. This example shows that a decrease in viscosity does not always translate into immediate mechanical failure; in PLA/PHB blends, crystallinity and morphology can partly compensate for rheological deterioration. However, the two-step thermal degradation behavior confirmed that PLA and PHB remained partly immiscible, so blend morphology must still be monitored during recycling.

A contrasting example is provided by Yarahmadi et al. [50], who examined PLA blended with conventional petroleum-based polymers, specifically PLA/HDPE and PLA/PC. Multiple extrusion did not strongly affect the elastic modulus of the blends, but it had opposite effects on elongation: PLA/HDPE showed improved elongation because repeated processing enhanced the dispersion of the PLA phase in HDPE, whereas PLA/PC lost elongation because degradation dominated. The study also showed why conventional-polymer blending is problematic for closed-loop PLA recycling. PLA/PC required a higher processing temperature, which promoted PLA degradation, and simulated post-consumer aging under humid conditions caused severe degradation of the PLA phase. Importantly, the PC phase also degraded, with BPA increasing from 0.13 to 2.3 µg BPA/g blend after accelerated aging. Therefore, this example supports the table’s caution that conventional-polymer blending may improve selected mechanical properties but can compromise PLA purity, compostability, safety profile, and subsequent recyclability.

## 6. Chemical, Hydrothermal, Thermochemical, and Biological Recycling Routes

PLA recycling studies differ widely in feedstock type, catalyst system, reaction severity, analytical reporting, and scale. Therefore, the main text presents a route-level decision summary in Table 2, while the complete study-by-study extraction table is provided as Supplementary Table S2 [57,58,59,60,61,62,63,64,65,66,67,68,69,70,71,72,73,74]. Table S2 includes detailed experimental information, such as reaction medium, catalyst, temperature, product yield, purity, racemization assessment, waste tolerance, repolymerization evidence, and scale. Table 2, therefore, condenses the available evidence into a decision-oriented overview of the main recycling routes, their evidence strength, advantages, limitations, and likely application roles.

Hydrolysis-based routes provide a direct approach for recovering lactic acid, but the required reaction severity varies considerably. Conventional hydrothermal treatment can achieve high PLA-to-lactic-acid conversion, especially at a range of 160–180 °C, but it generally requires pressurized water systems, long residence times, or elevated temperatures [57,58,59]. Microwave-assisted alkaline hydrolysis can substantially reduce reaction time, achieving high PLA degradation within minutes under the reported conditions. However, the use of NaOH and phase-transfer catalysts introduces additional considerations related to neutralization and wastewater management [60]. Subcritical-water treatment of real PLA waste indicates that post-consumer feedstocks can be processed, but yields are generally lower than those obtained from virgin PLA, suggesting that impurities and additives remain important barriers [58].

Alcoholysis provides a potentially milder and value-added alternative by converting PLA into alkyl lactates rather than directly into lactic acid. Zinc-based and dual-catalyst systems can produce methyl or ethyl lactate under comparatively moderate temperatures, and post-consumer PLA items have been converted to ethyl lactate even in the presence of unknown additives [61,62,63]. However, most alcoholysis studies report product formation by chromatographic quantification rather than isolated product purity, and repolymerization is usually discussed as a potential route through alkyl lactate-to-lactide conversion rather than experimentally demonstrated.

Less-studied chemical processing routes also deserve attention because they can convert PLA into functionalized oligomers or intermediates rather than only lactic acid. Glycolysis can yield hydroxyl-terminated lactic oligomers that may be used for lactide production, polyester modification, or polyurethane-type materials; alcohol–acidolysis combines molecular-weight reduction with functionalization; and aminolysis can generate amide-terminated lactic derivatives or oligomers. These routes broaden the valorization space for PLA waste, especially when monomer-grade closed-loop recovery is not realistic, but they remain less mature than hydrolysis and alcoholysis in terms of isolated product purity, mass balance, toxicity/safety assessment, and LCA reporting [21,55].

Thermochemical and thermocatalytic methods are mainly applied to mixed or real biodegradable consumer products such as PLA-based straws. CO_2_-assisted pyrolysis, sea-shell-derived catalysts, and MSW-bottom-ash catalysts can improve lactic acid or lactide recovery from PLA-containing straw waste, while also tolerating blended polymers such as PBAT and PBSA [64,65,66]. These approaches are potentially useful for mixed biodegradable waste streams and waste-derived catalyst integration, but monomer yields are generally lower than those of hydrolysis or optimized organocatalytic routes, and product purity and racemization are rarely evaluated.

Recent organocatalytic hydrolysis routes provide some of the clearest progress toward closed-loop PLA recycling. Diphenyl phosphate (DPP) enables solvent-free PLA hydrolysis with a small amount of water, producing low-molecular-weight oligo(lactic acid) that can be converted to lactide and repolymerized into PLA with reported high molecular weight [67]. The p-bis-nitrophenyl phosphate (p-BNPP) system further improves this concept by reducing the reaction time to 30 min at 160 °C, maintaining catalyst reusability over repeated cycles, tolerating commercial PLA products and mixed durable plastics, and demonstrating kilogram-scale conversion [69]. These organocatalytic studies also assess optical purity and report configuration retention, addressing a limitation of many earlier chemical recycling studies.

Biological and microbial routes provide a different valorization strategy rather than direct PLA-to-PLA recycling. Hydrolyzed PLA can be used as a carbon source for fermentation to PHB, microbial oil, or mixed carboxylates [57,69,70]. For example, PLA food-packaging waste hydrolyzed at 70 °C was fermented into C_2_–C_6_ carboxylates, with n-butyrate as the main reported product [68]. These studies indicate that PLA-derived lactate can enter biorefinery platforms, but they do not yet demonstrate direct repolymerization to PLA. Finally, PLA-specific binding peptides are not considered chemical recycling routes, but they are relevant as enabling tools for selective PLA recognition, sorting, and potential targeted degradation in mixed plastic streams [71].

Taken together, the evidence summarized in Table 2 and detailed in Supplementary Table S2 indicates that PLA chemical recycling is moving from model-polymer hydrolysis toward more realistic systems involving post-consumer waste, mixed plastics, additive-containing materials, reusable catalysts, and kilogram-scale demonstrations. Nevertheless, major gaps remain: many studies do not report isolated product purity, racemization is often not assessed, and direct closed-loop repolymerization is demonstrated only in a limited number of recent organocatalytic systems.

## 7. Environmental and Economic Assessments of PLA End-of-Life Routes

Table 3 summarizes the main environmental and economic trade-offs of PLA end-of-life routes, while detailed study-level LCA information, including system boundaries, functional units, geographical settings, energy mixes, and substitution assumptions, is provided in Supplementary Table S3 [75,76,77,78,79,80,81,82,83,84,85,86,87,88,89,90,91,92,93,94,95,96]. Across the reviewed studies, route ranking was strongly affected by whether the system credited recycled PLA, lactic acid, lactide, energy recovery, or other substituted products. Recycling routes generally performed best when recovered outputs realistically displaced virgin PLA or virgin monomer production, whereas composting, landfilling, and incineration provided weaker material-retention benefits [75,76,77].

The studies summarized in Table 3 indicate that mechanical recycling is the most favorable option when clean or efficiently sorted PLA is available and when recycled PLA can substitute virgin PLA at a meaningful quality-adjusted ratio. Maga et al. reported greenhouse-gas savings for post-consumer mechanical recycling because collection, preparation, and extrusion burdens were outweighed by avoided virgin PLA production [75]. Similarly, Moyaert et al. found mechanical recycling to have the lowest modeled climate-change impacts among PLA end-of-life pathways, with net-negative values across future scenarios, although the benefit depended on recyclate quality and future background systems [77].

Chemical recycling offers greater flexibility for degraded or mixed PLA streams, but its environmental advantage is more process-sensitive. Depolymerization to lactic acid, lactide, or regenerated PLA can reduce impacts when high-quality product recovery and substitution are achieved. However, high heat demand, solvent or catalyst use, incomplete recovery, and intensive purification can reduce or even reverse these benefits. Harmonization studies show that database choice, electricity mix, allocation method, and substituted product strongly affect the apparent performance of PLA depolymerization [76,77].

Composting, anaerobic digestion, and incineration have more limited circular value because they do not retain polymer material [78,79,80,81,82,83,84,85,86,87,88,89,90,91]. Composting may be suitable for certified compostable products or food-contaminated items when material recovery is impractical, but reviewed studies indicate limited climate benefit and possible burden shifting toward terrestrial ecotoxicity, eutrophication, land use, and infrastructure-related impacts [77,80,87,91]. Incineration with energy recovery is also context-dependent: it may appear more favorable under carbon-intensive energy systems, but its relative benefit declines as displaced electricity or heat becomes less carbon-intensive [77,78].

Product-level LCAs further show that PLA does not automatically outperform fossil-based or recycled conventional polymers. Outcomes depend on functional unit, product mass, reuse rate, recycled content, feedstock production, and real end-of-life infrastructure. For example, PET recycling or recycled LDPE packaging can outperform PLA-based alternatives when recycled content provides strong avoided-production credits [79,80]. Therefore, PLA end-of-life decisions should be based on route-specific LCA and techno-economic assumptions rather than on “bio-based” or “compostable” labels alone.

Finally, environmental benefits modeled for PLA recycling depend on upstream collection and sorting performance. NIR sorting studies indicate that PLA can be distinguished from common plastics, but detection and ejection are affected by transparency, thickness, degradation state, labels, fillers, and equipment settings [82,85]. Contamination studies also show that small amounts of PLA in PET, HDPE, PE, or mixed-polyolefin streams can downgrade recyclate quality [83,84,86]. Thus, LCA credits for recycled PLA are only realistic when waste systems can deliver sufficiently pure, dry, and application-suitable PLA streams.

## 8. Sorting, Contamination, and Infrastructure Challenges

**Figure 4:**
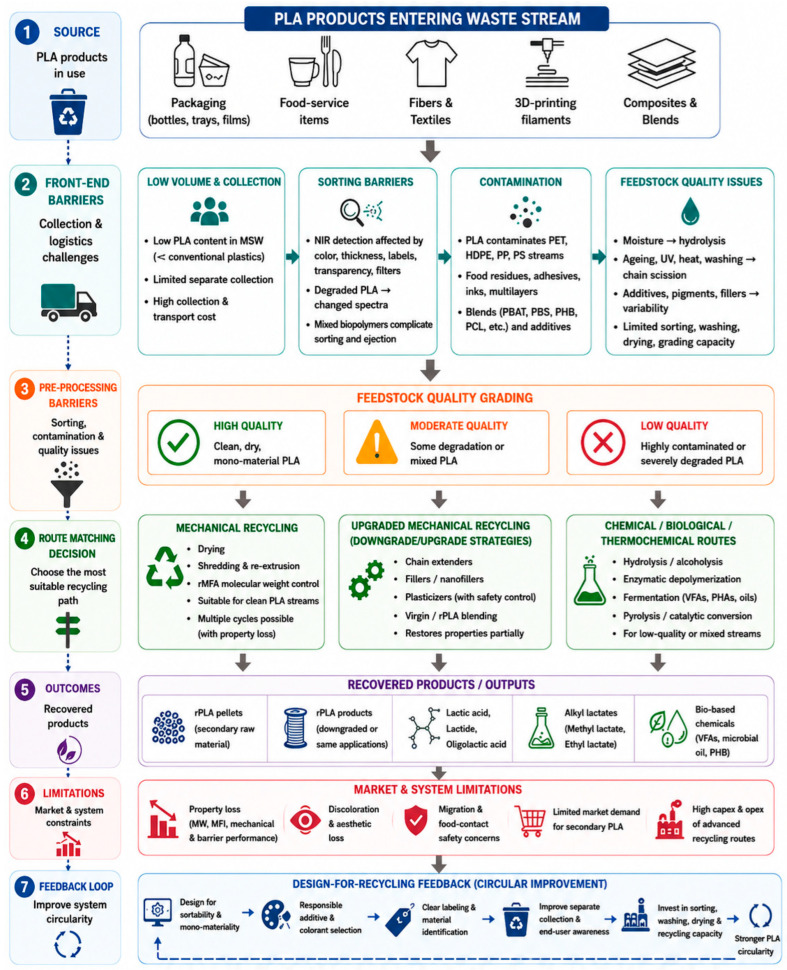
Conceptual scheme of sorting, contamination, and infrastructure barriers affecting PLA circularity. PLA recycling is constrained by low waste-stream concentration, incomplete collection, imperfect NIR sorting, contamination of conventional PET/HDPE/PP streams, moisture-driven hydrolysis, additive and blend diversity, and route-specific feedstock-quality requirements. Clean and dry mono-material PLA is most suitable for mechanical recycling, whereas moderately degraded PLA may require chain extenders, fillers, plasticizers, or virgin/recycled blending. Highly degraded, contaminated, filled, or blended PLA may be better directed to chemical, hydrothermal, alcoholysis, thermochemical, enzymatic, or fermentation-based routes. Final route selection should consider recyclate performance, food-contact safety, migration risk, market demand, energy mix, and environmental burden shifting.

The circular use of PLA depends not only on recycling chemistry, but also on the ability of waste-management systems to collect, identify, separate, dry, clean, and route PLA to an appropriate process. Although PLA is mechanically recyclable and chemically depolymerizable, post-consumer circularity remains limited by low waste-stream concentration, incomplete sorting, route-specific purity requirements, moisture sensitivity, formulation diversity, and limited markets for secondary PLA [75,76,77,78,79,80,81,82,83,84,85,86,87,88,89,90,91,92,93,94,95,96]. Figure 4 summarizes these barriers and illustrates why PLA recycling should be treated as a feedstock-quality and infrastructure challenge.

### 8.1. Low PLA Volume and Immature Collection Infrastructure

A major barrier to PLA recycling is its low concentration in municipal plastic waste relative to PET, PE, PP, and PS. This makes separate collection and profitable sorting difficult, even when PLA recycling is technically feasible [75,88]. As a result, PLA is often incinerated, landfilled, composted only under limited conditions, or mis-sorted into conventional recycling streams. The environmental benefits reported for PLA recycling therefore depend on regional collection networks, sorting capacity, and sufficient waste volumes to justify dedicated or route-specific infrastructure [75,76,77,81,88,90,92].

### 8.2. Sorting Requirements and PLA Identification

Sorting is essential both for generating usable PLA feedstock and for protecting established recycling streams. Near-infrared spectroscopy is currently the most relevant technology for identifying PLA in mixed packaging waste, but real-world performance depends on product geometry, transparency, thickness, backlight intensity, degradation state, labels, and fillers [82,85]. Therefore, sorting performance should be evaluated using product ejection success and downstream recyclate purity, not spectral classification alone. PLA-binding peptides and related bio-recognition tools remain at an early stage, but they suggest future options for more selective PLA detection or separation in mixed plastic systems [71,93].

### 8.3. Contamination of Conventional Recycling Streams

PLA should not be considered a harmless contaminant in conventional recycling. Because PLA volumes remain relatively low, misplaced PLA can enter PET, HDPE, PP, or mixed-polyolefin streams. Even small PLA or PLA-blend fractions can reduce elongation, impact strength, tensile performance, color stability, and clarity of conventional recyclates [83,84,86]. PET and HDPE streams are especially sensitive where food-packaging quality or optical performance is required [84,86]. Practical PLA infrastructure must therefore include contamination thresholds and sorting controls that protect both PLA recovery and existing recycling lines.

### 8.4. Route-Matched Infrastructure Demands

Different recycling routes require different feedstock qualities and facility designs. Mechanical recycling requires clean, dry, and compositionally homogeneous PLA, together with washing and drying systems that minimize hydrolysis during melt reprocessing [32,33,34,36,37,38,39,40,41,45]. Chemical and thermochemical routes can tolerate some degraded, filled, or mixed PLA streams, but they require reactors, catalyst or solvent management, gas handling where relevant, and purification systems for recovering lactic acid, lactide, or other platform chemicals [59,60,61,62,63,64,65,66,67,68,69,75]. Biological and hybrid biorefinery routes require pretreatment or pre-hydrolysis because solid PLA must first be converted into a microbial-accessible carbon source [57,69,70,94].

Formulation complexity further complicates routing. Blends such as PLA/HDPE or PLA/PC and additives such as chain extenders, plasticizers, and nanofillers can alter NIR signatures, melt rheology, degradation behavior, migration risk, and chemical-recycling compatibility [35,41,42,43,47,50]. Infrastructure planning must therefore account for both polymer identity and formulation history.

### 8.5. Design-for-Recycling Implications

PLA circularity is constrained less by the absence of recycling routes than by the lack of integrated systems able to connect product design, sorting, feedstock grading, and route selection. Reinforced, blended, colored, or chemically modified PLA should be evaluated not only for use-phase performance but also for NIR detectability, moisture sensitivity, depolymerization compatibility, migration safety, and future recyclability [35,39,40,44,47,48,49,50,51,52,53,56,72,73]. Practical implementation will require separate collection where feasible, formulation-aware sorting, strict drying control, and allocation of PLA streams to mechanical, chemical, biological, or lower-demand routes according to quality.

## 9. Decision Framework for Application, Safety, and Circular Use of Recycled PLA

**Figure 5:**
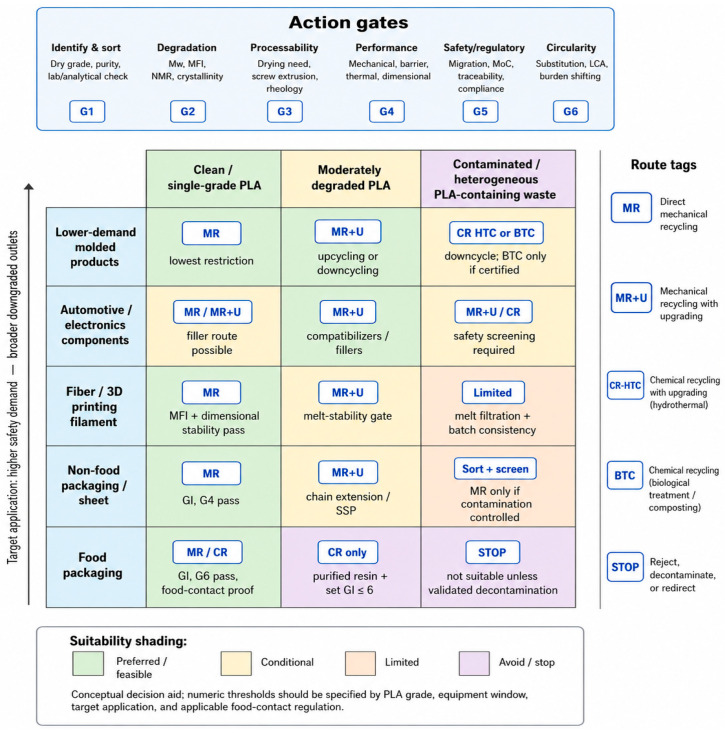
Application/safety decision map for recycled PLA streams. The map aligns feedstock quality with target application and route suitability. The right-hand inset summarizes the gate checklist used before assigning a recycled PLA stream to a target application.

Recycled PLA should be assigned to new applications according to evidence of feedstock quality, degradation state, processability, performance, safety, and circularity rather than according to its bio-based origin alone. Figure 5 presents this relationship as an application/safety map, and the gate checklist provides the minimum evidence needed before assigning a recycled PLA stream to target use.

### 9.1. Feedstock Quality as the First Decision Level

Feedstock quality is the first decision point. Clean, source-separated post-industrial or single-grade PLA offers the widest recycling window because polymer identity, additive history, and contamination risk are easier to control. These streams may be suitable for non-food packaging, sheet, filament, fibers, or molded products if molecular integrity and melt processability are retained. Food-contact use requires stronger evidence, including traceability, regulatory compliance, and migration testing. Moderately degraded PLA, including repeatedly processed, service-aged, or thermally aged material, requires closer evaluation. Chain scission, increased melt flow, reduced melt strength, and altered crystallinity can limit direct mechanical recycling. Upgrading by chain extension, solid-state polymerization, blending, compatibilization, or filler reinforcement may restore selected properties, but these interventions must also be assessed for migration, toxicity, additive accumulation, batch reproducibility, and future recyclability.

Contaminated or heterogeneous PLA-containing waste is the most constrained class. Mixed polymers, multilayers, pigments, food residues, unknown additives, and degradation products increase uncertainty in both performance and safety. Such streams should not enter food-contact applications unless validated sorting, decontamination, traceability, and migration evidence are available. If mechanical quality or safety cannot be restored, chemical or hydrothermal recycling may be more appropriate, provided that recovered products meet purity and stereochemical requirements.

### 9.2. Application Hierarchy and Route Selection

Target applications differ in their tolerance for variability and safety uncertainty. Food packaging is the most restrictive because mechanical and barrier performance must be supported by traceability, decontamination control, overall and specific migration testing, and assessment of non-intentionally added substances. Non-food packaging, sheet products, fibers, and 3D-printing filament can tolerate a wider range of recycled PLA qualities, but still require stable melt flow, dimensional stability, low moisture content, and reproducible processing. Composite and molded applications may accept broader property variation when reinforcement or compatibilization restores the required stiffness, heat resistance, or durability. Route selection follows the same hierarchy. Direct mechanical recycling is preferred for clean PLA with retained molecular integrity and processability. Upgraded mechanical recycling is appropriate when degradation is moderate and recoverable. Chemical or hydrothermal recycling is better suited to streams that cannot provide a reliable mechanical performance but still contain recoverable molecular value. Composting or biological treatment should be reserved for certified compostable products or application-specific cases where material recovery is technically impractical and environmental assessment supports the route.

### 9.3. Application/Safety Map

The application/safety map translates this hierarchy into a practical allocation tool. It aligns three feedstock classes—clean or single-grade PLA, moderately degraded PLA, and contaminated or heterogeneous PLA-containing waste—with applications ranging from food packaging to lower-demand molded products. Route tags indicate whether the most appropriate pathway is direct mechanical recycling, upgraded mechanical recycling, chemical or hydrothermal recycling, biological treatment or composting, or rejection/redirection. The map is intentionally conservative. Clean and traceable PLA has the greatest potential for high-value reuse, but only when the relevant gates are satisfied. Moderately degraded PLA may be directed to upgraded recycling, composites, or lower-demand products if performance and safety remain acceptable. Contaminated or heterogeneous streams should be excluded from high-safety applications unless decontamination and regulatory evidence are available. Where evidence is insufficient, the stream should be redirected rather than forced into a higher-value use.

### 9.4. Gate Checklist for Assigning Recycled PLA to Applications

Gate 1—Identity and sorting gate: Confirm PLA identity, grade consistency, and feedstock traceability. Streams with unresolved polymer contamination, multilayer structures, incompatible residues, unknown additives, or high food-residue burden should be further sorted, decontaminated, redirected, or rejected.

Gate 2—Degradation gate: Measure molecular weight or intrinsic viscosity, MFI/MFR, thermal transitions, and crystallinity. Direct mechanical recycling is appropriate only when molecular integrity and melt flow behavior remain within the processing window required by the target application.

Gate 3—Processability gate: Confirm drying adequacy, melt stability, melt strength, filtration behavior, and reproducible extrusion, injection molding, thermoforming, spinning, or filament production. Sheet, thermoforming, and filament applications require especially narrow control of melt behavior and moisture.

Gate 4—Performance gate: Verify that tensile strength, modulus, elongation at break, impact resistance, thermal stability, dimensional stability, barrier performance, and aging resistance meet the target application. Higher-value applications require narrower batch-to-batch variation.

Gate 5—Safety and regulatory gate: For food-contact use, recycled PLA must meet applicable regulatory requirements, including traceability, overall migration, specific migration, and assessment of non-intentionally added substances. Under EU food-contact plastic rules, the overall migration limit is 10 mg/dm^2^, corresponding to 60 mg/kg food under the standard conversion assumption. Streams containing unknown additives, colorants, degradation products, or contaminants should not be assigned to food-contact use unless safety is demonstrated.

Gate 6—Circularity and burden-shifting gate: Confirm that the selected route provides a circularity benefit relative to realistic alternatives. This assessment should include substitution ratio, energy demand, energy mix, water use, toxicity, land use, ecotoxicity, product lifetime, additive accumulation, and future recyclability [95].

### 9.5. Practical Decision Logic

The framework assigns recycled PLA to the highest-value application supported by its evidence base, not simply to the highest-value application in principle. Clean and well-characterized PLA should first be evaluated for direct mechanical recycling [96]. Moderately degraded material may justify upgrading if safety and future recyclability are not compromised. Highly degraded, contaminated, or heterogeneous streams should be redirected to chemical, hydrothermal, biological, composting, or lower-demand routes according to product quality and environmental performance.

## 10. Conclusions

This review shows that PLA recycling is a feedstock-quality-dependent circular-materials challenge rather than a simple choice among end-of-life routes. Clean, dry, and compositionally known PLA streams are the strongest candidates for mechanical recycling. In contrast, service-life aging, moisture exposure, repeated thermal and shear history, and polymer or additive contamination accelerate molar-mass loss, melt flow changes, crystallinity shifts, and property deterioration.

Upgrading strategies can extend the usable window of recycled PLA, but their suitability depends on the degradation state and target application. Chain extenders, solid-state polymerization, compatibilizers, plasticizers, blends, fibers, and mineral fillers can restore or compensate for selected properties, but they also introduce safety, migration, sorting, and subsequent-recyclability questions that must be evaluated together with performance recovery.

Chemical, hydrothermal, thermochemical, enzymatic, and hybrid biorefinery routes expand the options for PLA streams that are unsuitable for high-quality mechanical recycling. Their circular value depends on product yield, purity, stereochemical retention, catalyst or solvent management, energy demand, contaminant tolerance, and evidence that recovered products can re-enter polymer or platform-chemical production. Biological routes are promising for valorization, but they generally require pretreatment, sufficient residence time, and suitable microbial or enzymatic systems.

The environmental evidence does not support a universal hierarchy for PLA end-of-life management. Mechanical recycling often gives favorable climate and resource results when high-quality substitution is achieved, but this advantage depends on collection efficiency, sorting quality, transport, energy mix, recyclate quality, and realistic substitution factors. Composting, chemical recycling, and energy recovery may be appropriate under specific feedstock and infrastructure conditions, but their benefits should be assessed together with burden shifting in land use, water use, toxicity, eutrophication, and conventional-recyclate contamination.

Future studies should report waste history, moisture and drying conditions, processing severity, molecular-weight or viscosity changes, stereochemical quality, additive composition, migration and toxicity evidence where relevant, application-specific property retention, and LCA boundaries. Route-matched sorting, feedstock grading, and application-specific safety gates are needed to move PLA from a disposal-oriented bioplastic toward a quality-controlled circular material.

The practical conclusion of this review is therefore conservative but circular: retain PLA at the highest material value only when feedstock quality and safety evidence support that use, and redirect unsuitable streams to route-matched chemical or biological recovery rather than relying on biodegradability as a universal end-of-life solution.

## Figures and Tables

**Table 1:** Compact decision-oriented summary of recycled PLA upgrading strategies. Detailed study-level evidence is provided in Supplementary Table S1.

Strategy	Primary Benefit	Key Limitation/Safety Gate	Implication for Subsequent Recyclability	References
Additive-free repeated reprocessing/baseline control	Provides a reference for judging whether upgrading is necessary; clean pre-consumer PLA may tolerate several controlled cycles.	MFI/MFR rise, viscosity and molecular-weight decline, discoloration, and brittleness can accumulate; degradation products should be checked for sensitive packaging uses.	Appropriate mainly for clean, dry, well-sorted PLA; recyclability declines as chain scission accumulates.	[32,33,36,38]
Epoxy-functional chain extenders	Restore molar mass, melt strength, viscosity, thermal stability, and processability by reacting with PLA end groups.	Dose-sensitive; epoxides can react with carboxyl and hydroxyl end groups and form secondary, less reactive hydroxyls. Acid/hydroxyl number, moisture, residence time, and dosage control are required.	Positive when dosage is controlled; over-branching, gel formation, or high viscosity may reduce predictable remelting, filtration, and later depolymerization.	[35,41,43,55,56]
Isocyanate chain extenders/polyester-urethane formation	Can increase molar mass, melt strength, toughness, or durability through urethane linkage formation.	Moisture-sensitive chemistry; residual isocyanates and processing safety must be controlled. Food-contact use requires specific authorization, extractables, and migration evidence.	May be useful for durable non-food products, but urethane-containing structures can complicate closed-loop PLA purity and chemical-recycling behavior.	[55]
Oxazoline chain extenders/polyester-amide formation	Can react with carboxyl end groups and introduce amide-containing linkages that improve molecular integrity.	Less reported in recycled PLA studies; dosing, residual oxazoline, hydrolysis behavior, and extractables require validation.	Potentially useful in controlled reactive extrusion, but altered linkage chemistry should be considered before repeated recycling or depolymerization.	[55]
Multifunctional chain extenders	Joncryl-type or epoxy/dianhydride systems recover melt viscosity and stabilize post-process scrap or PLA/PBS formulations.	Narrow processing window; excessive functionality may reduce flow or create branched/crosslinked structures. Migration/toxicity evidence is often missing.	Can extend useful recycling life, but excessive branching may make future recyclability less predictable.	[41,42,49]
Organic peroxides	Low doses can promote radical branching/crosslinking and improve viscosity, thermal stability, or hardness.	Competing chain scission, gel formation, residual peroxide/by-products, and brittleness are possible if overdosed.	Potentially useful only under controlled low-dose conditions; radical-modified PLA may be harder to recycle consistently.	[41]
Solid-state polymerization/post-condensation	Rebuilds molecular weight below the melting point without adding a second polymer phase.	Slow, moisture-sensitive, and suitable mainly for clean, dry, sorted PLA; residual monomer/catalyst/volatiles should be checked.	Favorable for closed-loop PLA because chemistry remains mostly PLA, although crystallinity changes may alter later processing.	[32,56]
Plasticizers	Improve flexibility, elongation, chain mobility, and film-forming ability in brittle rPLA.	May reduce stiffness, heat resistance, dimensional stability, and barrier performance; migration is a key limitation.	Can complicate later recycling because plasticizers may migrate, volatilize, or accumulate; re-formulation may be required.	[35,51,54]
Compatibilizers	Improve interfacial adhesion in PLA/fiber or PLA/polymer blends.	Increase chemical complexity and can alter MFI, crystallinity, hydrolysis, biodegradation, sorting behavior, and extractables.	Positive when they prevent phase separation; negative if they create irreversible networks or poorly defined stream composition.	[49,50,51,52,53]
Fibers and mineral fillers	Natural fibers, HNTs, chitosan, silk fibroin nanoparticles, talc, TiO2, and related fillers can improve stiffness, strength, crystallinity, thermal resistance, or barrier behavior.	Agglomeration, reduced elongation, moisture sensitivity, color change, abrasion, and nanofiller migration/inhalation questions may occur.	Repeated recycling may concentrate fillers and worsen dispersion; filler-rich streams may need separate sorting.	[44,47,51,52,53]
Biodegradable polyester blends: PBAT, PHB, PBS, PCL, aPHA	Can improve toughness, ductility, crystallization, flexibility, or barrier behavior depending on blend morphology.	Immiscibility and phase separation are common; blend-specific migration/extractables evidence is needed for packaging.	Moderate to good when morphology remains stable; controlled ratios are essential for later recycling.	[37,38,39,40,48,49,53]
Virgin/rPLA or conventional-polymer blending	Virgin PLA can dilute degradation; conventional polymers may improve selected properties or reduce cost.	Conventional-polymer blending compromises PLA purity and compostability; immiscibility and high-temperature degradation can occur. BPA was detected in aged PLA/PC blends.	Virgin/rPLA blending is favorable for controlled industrial scrap; conventional-polymer blending is problematic for closed-loop PLA.	[32,34,36,46,50]

**Table 2:** Compact route-level decision summary of chemical, hydrothermal, thermochemical, organocatalytic, and biological PLA recycling strategies. Detailed experimental extraction is provided in Supplementary Table S2.

Route	Main Recovered Product/Function	Evidence Strength	Main Advantage	Key Limitations and Best Current Role
Hydrolysis/hydrothermal treatment	Lactic acid from neat PLA, PLA waste, or post-consumer PLA	High for model PLA; moderate for real waste	Direct monomer recovery; high conversion possible at a range of 160–180 °C.	Requires pressurized water, residence time, and purity/racemization control; best for relatively clean PLA streams.
Microwave-assisted alkaline hydrolysis	Lactic acid/lactate salts	Moderate	Very fast depolymerization under reported conditions.	NaOH, neutralization, phase-transfer additives, and wastewater burdens must be assessed.
Alcoholysis/methanolysis/ethanolysis	Methyl or ethyl lactate from PLA and post-consumer items	Moderate to high	Often milder than hydrolysis; produces value-added alkyl lactates and can tolerate some real-waste additives.	Product isolation, purity, catalyst recovery, and closed-loop conversion back to lactide/PLA require stronger evidence.
Glycolysis, alcohol-acidolysis, and aminolysis	Hydroxyl-, ester-, acid-, or amide-functionalized lactic oligomers/intermediates	Emerging to moderate	Broadens PLA valorization beyond lactic acid/lactide; can produce functional oligomers for polyester or polyurethane-type materials.	Less studied than hydrolysis/alcoholysis; product purity, toxicology, mass balance, and LCA reporting are often incomplete.
Thermochemical/catalytic pyrolysis	Lactide, lactic acid, and other oxygenates from PLA-containing consumer products or biodegradable blends	Moderate	Can treat heterogeneous PLA-containing biodegradable waste and integrate waste-derived catalysts.	Lower selectivity; racemization, catalyst residues, and product purity are rarely fully assessed.
Organocatalytic closed-loop depolymerization	Oligomers, lactide, and repolymerizable intermediates	High in recent studies	Reusable catalysts, optical-purity assessment, kg-scale evidence, and repolymerization shown in selected systems.	Still limited number of systems; real-waste validation and scale-up need further work.
Biological/microbial valorization	PHB, microbial oil, carboxylates, biomass, or other biorefinery products from hydrolyzed PLA	Moderate	Converts PLA-derived lactate carbon into value-added bioproducts.	Not direct PLA-to-PLA recycling; residence time, pretreatment, and organism/enzyme selection are critical.
PLA-specific binding/enzymatic-enabling tools	Selective PLA recognition, sorting support, or enhanced targeted degradation in mixed plastics	Emerging	Supports detection, pre-sorting, and targeted depolymerization.	Platform-enabling evidence rather than a standalone recycling route.

**Table 3:** Compact decision-oriented summary of environmental and economic findings for PLA end-of-life routes. Reported climate-change values are not directly interchangeable because reviewed studies used different system boundaries, functional units, geographical settings, allocation methods, energy mixes, substitution credits, and recyclate-quality assumptions; detailed LCA extraction is provided in Supplementary Table S3.

End-of-Life Route	Representative Climate/Economic Signal	Substitution or Credit Assumption	Key Burden Shifting/Implementation Issue	Decision-Oriented Interpretation
Mechanical recycling	Prospective modeling reported the lowest climate-change impact among modeled PLA EoL routes (about −1.22 to −0.67 kg CO2-eq/kg PLA). Eco-efficiency studies also favored 100% mechanical recycling when high-quality substitution was assumed.	Benefits depend on quality-adjusted replacement of virgin PLA by recyclate.	Requires collection, sorting, washing, drying, extrusion, and sufficient market volume; water use can increase in some scenarios.	Preferred for clean, dry, compositionally known PLA streams when recycled PLA can realistically substitute virgin PLA.
Chemical recycling with material recovery/repolymerization potential	Harmonized depolymerization studies reported credited GWP values of about −2869 to −1378 kg CO2-eq/Mg PLA waste; second-generation PLA LCA showed GWP reduction when chemical recycling was integrated.	Credits depend on whether recovered lactic acid, lactide, regenerated PLA, or other products replace virgin products.	Sensitive to heat, electricity mix, catalysts, solvents, purification, yield, allocation, and database choice.	Suitable for degraded, mixed, or lower-quality PLA when mechanical recycling cannot meet performance requirements and product recovery is robust.
Chemical recycling without repolymerization or weak substitution	Prospective modeling showed impacts up to +0.58 kg CO2-eq/kg PLA when closed-loop replacement was absent or weak.	Lower-value products provide smaller avoided-production credits.	High heat demand, incomplete recovery, solvent/catalyst inputs, and purification can offset benefits.	Should not be assumed superior; requires route-specific LCA and techno-economic validation.
Industrial composting/biological treatment	Generally weaker climate and circularity benefits than recycling because material value is not retained.	Usually provides little or no virgin-material substitution credit.	Potential burden shifting to terrestrial ecotoxicity, eutrophication, land-related impacts, transport, and infrastructure; requires certified industrial composting conditions.	Appropriate for certified compostable, food-contaminated, or non-recyclable items only when material recovery is impractical.
Incineration with energy recovery	Can appear competitive under carbon-intensive electricity but loses advantage as energy systems decarbonize.	Energy credit depends on displaced electricity/heat.	Releases stored biogenic carbon and loses embedded material value.	Residual route for non-recyclable fractions rather than a preferred circular strategy.
Product-level PLA/bioplastic systems and recycled conventional-polymer comparators	PLA does not automatically outperform fossil-based or recycled conventional polymers; PET recycling and recycled LDPE can outperform PLA alternatives in some functional units.	Benefits depend on product mass, reuse rate, recycled content, feedstock, and real EoL infrastructure.	Bio-based PLA can shift burdens to agricultural land, water, fertilizer, eutrophication, ecotoxicity, and feedstock production.	Product decisions should be based on functional unit, reuse/recycling assumptions, safety, and actual infrastructure rather than bio-based or compostable labels alone.

## Data Availability

No new data were created or analyzed in this study. Data sharing is not applicable to this article.

## Supplementary Materials

The following supporting information can be downloaded at https://www.mdpi.com/article/10.3390/polym18141731/s1, Table S1: Mechanical recycling of neat and post-consumer PLA. NR: not reported or not measured; PC: post-consumer; CE: chain extender; DCP: dicumyl peroxide; HNT: halloysite nanotube; IW: industrial waste; TS: tensile strength; YM: Young’s modulus; EB: elongation at break; MFI/MFR: melt flow index/rate; OP: oxygen permeability; WVP: water-vapor permeability; WVTR: water-vapor transmission rate; Table S2: Chemical and hydrothermal recycling routes; Table S3: Environmental and, where available, economic assessments of PLA/bioplastic recycling and end-of-life options. Abbreviations: MR, mechanical recycling; CR, chemical recycling/depolymerization; MSWI, municipal solid-waste incineration; AD, anaerobic digestion; FU, functional unit; GWP, global warming potential; EoL, end of life; NR, not reported; N/A, not applicable.
