# Supplementary material for: Recycling of Poly(lactic acid): From Molecular Degradation to Circular End-of-Life Strategies
Source: Polymers (Basel). 2026 Jul 15;18(14):1731. doi: 10.3390/polym18141731 (PMC13418005; doi:10.3390/polym18141731)
Supplement: Supplementary file 1 [file polymers-18-01731-s001.zip › polymers-4434596-supplementary.pdf]

Supplementary Information

Table S1. Mechanical recycling of neat and post-consumer PLA. NR: not reported or not measured; PC: post-consumer; CE: chain extender; DCP: dicumyl peroxide; HNT: halloysite nanotube; IW: industrial waste; TS: tensile strength; YM: Young's modulus; EB: elongation at break; MFI/MFR: melt-flow index/rate; OP: oxygen permeability; WVP: water-vapor permeability; WVTR: water-vapor transmission rate.

Waste source and PLA grade	Simulated or real post-consumer material	Service-life treatment	Drying conditions	Recycling cycles	Processing temperature	Molecular-weight change	MFI or viscosity	Crystallinity	Mechanical-property retention	Barrier-property changes	Proposed application	Reference
Commercial PLA water bottle, Cabreiroá; bottle-grade PLA	Simulated secondary PC material	UVB 40 h; 50 °C for 468 h; water at 25 °C for 240 h; NaOH/Triton washing at 85 °C for 15 min	40 °C for 72 h after washing	One extrusion after simulated PC aging	Extrusion at 180 °C, 100 rpm, 3 min	Mw decreased from 186.7 to 158.7 kg/mol after aging/washing and to 131.6 kg/mol after reprocessing	MFI increased from 14.54 to 15.42-15.53 g/10 min	Xc increased from 4.2% to 28.1% after PC simulation, then decreased to 10.7% after reprocessing	TS and YM largely retained; EB decreased from 11.4% to 8.1%	OP and WVP increased; reprocessed bottle showed poorer barrier performance than original bottle	Lower-barrier food packaging; may be unsuitable for bottle-to-bottle recycling without barrier correction	[34]
Commercial PLA water-bottle preforms, Cabreiroá	Real commercial preforms; primary recycling; not PC	None; clean preform material	Vacuum dried at 40 °C overnight before extrusion; drying repeated between cycles	Six consecutive extrusion cycles	Extrusion at 180 °C, 100 rpm, 3 min; cast films processed at 210 °C	Mw decreased from 186.7 to 113.9 kg/mol after six cycles, about 40% loss	MFI increased from 10.27 to 15.30 g/10 min; maximum 15.81 g/10 min after four cycles	Xc increased from 6.9% to 39.5% after six cycles	NR	OP and WVP worsened, especially at high RH; high MFI made later-cycle film casting difficult	Low-humidity, non-oxidation-sensitive food packaging; water-bottle reuse would require additional validation	[32]

Simulated PC PLA bottle upgraded with OLA plasticizer or OLA-based CE	Simulated PC bottle material	UVB 40 h; 50 °C for 468 h; water at 25 °C for 240 h; NaOH/Triton washing	40 °C for 72 h after washing	One extrusion with additive after PC simulation	Extrusion at 180 °C, 100 rpm, 3 min; compression at 160 °C; injection at 180 °C	Mw decreased from 155.4 kg/mol in bottle PLA to 131.3 kg/mol after PC simulation and 119.5 kg/mol in rPLA; CE-modified rPLA remained around 100-101 kg/mol	NR	First-heating Xc: rPLA 2.2%; plasticized rPLA 6.2-11.8%; CE-rPLA 0.4-9.5%	CE retained TS around 64 MPa with EB around 40%; 30% plasticizer increased EB but reduced TS and stiffness	WVP changed moderately; 30% plasticizer increased WVP at high RH; CE showed limited barrier penalty	Food packaging; CE-modified rPLA more suitable than highly plasticized rPLA for food-contact use	[35]
Ingeo 2003D packaging-grade PLA; simulated PC and severely hydrolyzed PLA	Simulated PC and severely degraded residue	Simulated PC: UVB 40 h, 50 °C for 468 h, water at 25 °C for 240 h, NaOH/Triton washing; hydrolyzed residue: water at 60 °C for 5 days	Vacuum dried at 85 °C for 2 h before melt processing	Initial film preparatio n plus one recycling extrusion with CE or DCP	Twin-screw processing at 125-190 °C; compression molding at 190 °C	PLAV Mw about 153 kg/mol; simulated recycled PLA Mw about 146 kg/mol; hydrolyzed PLA showed larger viscosity loss	Intrinsic viscosity decreased by about 17% in simulated recycled PLA; additive response depended on dose and degradation level	Recycled materials remained mostly amorphous; Xc about 0-4%	Microhardness decreased after recycling; CE or DCP partially recovered hardness depending on dose and degradation level	NR	Additive-assisted upgrading of recycled PLA for packaging, agriculture, and automotive applications	[41]
Mechanically recycled PLA with chitosan or silk fibroin nanoparticles	Simulated PC and hydrolyzed recycled PLA; filler-assisted recycling	Accelerated aging and severe hydrolysis routes used before recycling	NR	One recycling compound ing step with organic fillers	Melt compounding and film formation; exact processing profile NR in table context	Intrinsic viscosity depended on filler type and degradation level; fillers reduced reprocessing degradation in severely hydrolyzed PLA	Viscosity improved in selected filler systems	Fillers acted as nucleating/structural modifiers; Xc varied with filler type	Selected fillers improved hardness and selected mechanical/thermal properties	OP and WVTR could decrease with well-dispersed silk fibroin nanoparticles; chitosan effect depended on molecular weight and dispersion	Bio-based filler strategy for improving recycled PLA films	[44]

Ingeo 3251D injection-molding PLA; runners/rejected parts reprocessed as RPLA	Real pre-consumer injection waste; not PC	150 days in outdoor weathering, seawater, or river water	NR	One reprocessing from injection-molding waste, followed by aging	Injection molding; temperature NR	NR	NR	Xc NR; Tg and Tm showed the largest decrease after river-water aging	RPLA TS decreased from 29.5 MPa to 17.5 MPa outdoors, 14.9 MPa in seawater, and 7.1 MPa in river water; impact and hardness losses exceeded VPLA losses	NR	Durability assessment for outdoor and aquatic service; stabilization needed for wet exposure	[45]
NatureWorks 2002D thermoforming-grade PLA; virgin/reprocessed PLA blends	Simulated primary recycling of clean production scrap	None	PLA dried at 80 °C for 2 h; dried before each processing cycle; films dried at 50 °C under vacuum for 24 h	Up to two injection reprocessing cycles; blends with 40-80 wt% reprocessed PLA	Injection at 160-200 °C; blend extrusion at 185-195 °C; compression at 170 °C	NR	DMTA showed similar or slightly improved storage modulus in blends	Amorphous PLA structure mostly preserved	Mechanical blend effectiveness increased; service-relevant viscoelastic performance remained close to virgin PLA	NR	Recycling of production scraps for thermoforming and packaging; energy valorization as final route	[46]
Luminy LX175 PLA-based formulation with PBS, talc, and Joncryl CE	Post-process/pre-consumer material; PLA-based blend, not neat PLA	None	50 °C for 7 h under vacuum before compounding; 60 °C for 8 h before cast extrusion	Three compounding cycles; cast extrusion with 0-100% post-process material	Twin-screw compounding and cast extrusion; formulation-dependent temperature profiles	MFR increase indicated molecular-weight loss; CE during cast extrusion recovered flow behavior	MFI increased with cycles; CE reduced MFI toward original compound	Reprocessing increased crystallinity; CE reduced crystallinity by increasing chain length and limiting crystallization	Repeated cycles did not significantly change maximum stress, strain at break, or YM; CE improved EB in 100% recycled slabs	NR	Cast-extruded and thermoformed sheets; recovery of trimming scrap	[42]
NatureWorks 2003D PLA; rPLA chain-extended with RAFT GS-SAN copolymers	Simulated mechanical recycling; not PC	No service aging; thermo-mechanical degradation by extrusion	Dried before extrusion; exact drying temperature NR	Two extrusion cycles to obtain rPLA, followed by extrusion with 2 wt% CE	Two-cycle extrusion at 200 °C; CE extrusion with 80-190 °C profile	Mn decreased from 110 to 63 kg/mol after recycling; CE restored Mn up to about 104 kg/mol	Complex viscosity decreased from about 2300 to 330 Pa·s; CE restored it to about 2000 Pa·s	Xc increased from 20% to 22% after recycling and to 24-29% after CE addition	Direct tensile data NR; rheology indicated improved melt strength and processability	NR	Improved melt processing of rPLA, especially packaging-oriented applications	[43]

Mixed rPLA from failed 3D-printing objects and leftover filament spools; rPLA/HNT composites	Real clean PLA waste; not formal PC	Sorted, washed with distilled water, air-dried, shredded	rPLA flakes dried at 60 °C for 6 h; rPLA/HNT dried at 60 °C for 12 h	One melt-compounding and injection-molding route; HNT 1-5 wt%	rPLA extrusion at 170-190 °C; composite extrusion at 160-190 °C; injection at 185-195 °C	NR	rPLA MFI about 6 g/10 min	Xc increased from 57.49% in rPLA to 59.22% with 5 wt% HNT	TS increased from 42.98 MPa to 49.39 MPa at 2 wt% HNT; flexural strength improved by 22% at 3 wt% HNT; compressive properties increased at 5 wt% HNT	OP/WVP not measured; thermal barrier effect improved by HNT, with higher degradation temperature and char residue	Packaging, rigid housings, automotive interiors, and structural bioplastics	[47]
PLA/PHB blend, 70/30 wt%; PLA 4044D and PHB P226	Simulated multiple mechanical recycling of blend; not PC	None	PLA and PHB dried separately at 60 °C overnight; pellets dried at least 2 h before each reprocessing cycle	Five extrusion recycling cycles	Twin-screw extrusion 60-200 °C; screw 50 rpm; injection molding 170-185 °C	Direct Mw not central in table context; degradation inferred from viscosity decrease and morphology changes	Viscosity decreased significantly with recycling; MFI/rheology indicated chain scission and improved phase dispersion	Xc increased with reprocessing; PHB droplets acted as nucleating agents for PLA	Tensile properties were not significantly affected; impact behavior maintained because crystallinity and morphology counterbalanced viscosity loss	Barrier properties not directly evaluated in recycling section	Sustainable packaging applications using recyclable PLA/PHB blends	[39]
PLA/aPHA blends: 100% PLA, 90% PLA/10% aPHA, 75% PLA/25% aPHA	Simulated reprocessing of neat PLA and PLA/aPHA blends; not PC	None	Resin pellets dried at 45 °C for 24 h before testing/processing	Up to five heat cycles in extrusion; injection and compression molding evaluated	Extrusion 180-200 °C; injection molding around 210 °C; compression molding around 190 °C	NR	MFR increased with heat cycles, especially in neat PLA; complex viscosity decreased after repeated heat cycles	PLA crystallinity increased with heat cycles; aPHA suppressed or modified PLA crystallization depending on blend ratio	75% PLA/25% aPHA improved toughness compared with PLA; repeated processing decreased viscosity but blends remained processable	Barrier properties NR	Thermoformed packaging; recyclable PLA/aPHA blends for applications requiring improved ductility	[48]

PLA/PBS blends with high secondary raw material; formulations with PBS, talc, Joncryl, EBS	Simulated mechanical recycling with secondary raw material; not PC	None	Pellets dried at 45 °C for 6 h before cast extrusion	Industrially inspired sequence with up to three extrusion cycles; 45% secondary material used in later cycles; CE addition tested	Twin-screw compounding and cast extrusion; compounding around 145-190 °C depending on formulation; cast extrusion around 170-190 °C	MFR trends indicated polymer-chain degradation and partial recovery by CE	MFR decreased or stabilized when Joncryl was used; melt pressure increased with CE, indicating higher melt viscosity	PLA crystallinity changed with formulation and CE; higher chain extension tended to limit crystallization	High PBS content maintained flexibility; CE improved stability when PLA was the main phase	NR	Film/cast-sheet applications using high amounts of secondary PLA/PBS raw material	[49]
Ingeo 3025D PLA; injection-molding grade; also mixed with industrial PLA/PBAT waste containing 85 wt% PLA and 15 wt% PBAT	Simulated industrial mechanical recycling; not PC	None	80 °C overnight before injection molding; DSC specimens dried at 40 °C for at least 12 h	Up to 10 injection-molding/grinding/remolding cycles; PLA/PBAT IW blends also prepared	Injection molding; barrel 195-175 °C, mold 25 °C	Direct Mw not reported; degradation inferred from MFR increase and crystallization behavior	MFR increased substantially; about 18 g/10 min after first cycle and 277 g/10 min after seventh cycle; cycles 8-10 not measurable under applied conditions	Average injection-molded crystallinity remained low, around 5%; cold crystallization/melting peaks increased with recycling	PLA stable up to 5-6 cycles; TS decreased from 51.73 MPa after first cycle to 27.94 MPa after tenth cycle; deformation at break decreased from 2.42% to 0.84%	NR	Industrial recycling of injection-molded PLA and mixed PLA/PBAT waste streams; feasible mainly up to about 5-6 cycles	[38]
Pre-consumer industrial PLA bottle waste	Real pre-consumer industrial waste; additive-free; not PC	None; waste used as received	Milled material dried at 75 °C between cycles; no plasticizer or other additive	10 processing cycles	Twin-screw extrusion 165-180 °C; injection molding 165-180 °C; mold 45 °C; cooling 20 s	Mw not directly reported; chain scission inferred from increasing MFI and decreasing viscosity	MFI increased from about 8.7 to 13.9 g/10 min; viscosity decreased progressively	Xc remained low and variable, roughly 1-13%; characteristic crystallinity remained below 20%	Flexural and tensile properties remained largely stable; tensile strength about 68.2 MPa after cycle 1 and 67.1 MPa after cycle 10	NR	Closed-loop mechanical recycling of clean industrial PLA waste; aesthetic/color-sensitive use may require color stabilization	[33]

Total Corbion PLA LX175; neat PLA compared with PBAT, PBS, PBAT/PBS, and PBAT/TPS	Simulated mechanical recycling; not PC	None	80 °C for 8 h before extrusion; pellets dried again after pelletization	Up to seven cycles planned; neat PLA could not be reprocessed beyond sixth cycle because of low melt strength	Twin-screw extrusion 120-180 °C; injection molding 130-180 °C	Direct Mw not reported; degradation inferred from MFI increase and reduced melt strength	Final PLA MFR after last cycle about 53.2 g/10 min; PLA not processable beyond this condition	Cold-crystallization temperature decreased with cycles; Hc and Hm remained similar	PLA TS was 58.7 MPa with 8% strain at break after first cycle; TS decreased about 10% by sixth cycle; strain at break decreased about 44% by second cycle	NR	Limited repeated recycling of neat PLA; PBAT/TPS-type systems showed better reprocessing stability	[37]
NONOILEN IM 3056-2 PLA/PHB blend, PLA/PHB 45/55 by weight	Simulated material recycling of PLA/PHB blend; not neat PLA and not PC	None	NR	11 single-screw extrusion passes	Single-screw extrusion profile 170-190-185-180 °C; screw speed 55 min^{-1}; residence time 58 s per cycle	GPC showed decreasing molecular weight and decreasing polydispersity with processing	Complex viscosity decreased with extrusion time; relative viscosity after 4 min remained about 70-80% of initial value	Thermal properties and crystallite melting behavior changed minimally; no major crystallinity deterioration	Tensile strength remained broadly stable, about 23-35 MPa; EB decreased mainly in early cycles	Not directly measured; stable thermal/crystallinity behavior suggested no major barrier deterioration	Material recycling of PLA/PHB blends for applications not requiring high flexibility	[40]
PLA 2003D; compared with Bioflex, Solanyl, and PHBV	Simulated mechanical recycling; not PC	None	80 °C for 24 h before extrusion; extruded pellets dried at 60 °C for 24 h before compression molding	Five extrusion cycles	Twin-screw extrusion for PLA: 152-168 °C with die at 160 °C; screw 180 rpm; compression molding at 160 °C for 5 min	Mw decreased from 203,500 to 44,149 g/mol after five cycles, about 78% reduction	MFI increased from 10.60 to 18.20 g/10 min	Xc decreased from 33.17% to 7.01%	Flexural strength decreased slightly from 77.7 to 75.0 MPa; flexural modulus from 2.5 to 2.2 GPa; impact strength from 7.7 to 7.0 kJ/m^2; storage modulus decreased about 7%	NR	Benchmark for PLA degradation during reprocessing; stabilization or blending needed for repeated recycling	[36]

Ingeo 3051D PLA and commercial PLA/HDPE and PLA/PC blends	Simulated pre-consumer reprocessing and post-consumer aging/recycling	Accelerated aging at 50 °C and 90% RH for 7 weeks, estimated as roughly one year of service	Materials kept dry or vacuum-dried before each extrusion and melt pressing	Up to six consecutive extrusions; post-consumer recycling simulated after aging	Extrusion melt temperature about 195 °C for PLA and PLA/HDPE; about 240 °C for PLA/PC; compression at 205 °C for PLA and PLA/HDPE and 250 °C for PLA/PC	Direct Mw not reported; degradation inferred from MFI increase, crystallization, and aging response	Pure PLA MFI increased almost linearly with extrusion number	Multiple processing induced PLA crystallization; Tg and impact strength of pure PLA were not strongly affected by processing	Pure PLA impact strength was not affected by multiple extrusion; blend modulus remained almost unchanged; elongation changed depending on blend type; pure PLA showed marked degradation after aging	NR	Durable biobased applications where production waste may be reprocessed; PLA/PC resisted aging better than pure PLA but still degraded	[50]
Recycled PLA from Cargill Dow blended with sisal fibers; PLA-g-AA/SF composites	Recycled PLA-based composite; contextual row, not recycling-cycle study	Soil burial for biodegradation; no pre-processing service-life aging	Sisal fiber dried and vacuum-dried; composites conditioned at 50 ± 5% RH before mechanical testing	No repeated recycling cycles; one melt-compounding/compression-molding route	PLA-g-AA grafting at 190 ± 5 °C; composite mixing at 180-190 °C for 25 min; hot pressing at 180 °C	Recycling-induced Mw change NR	Melt torque decreased with increasing sisal fiber content; PLA-g-AA/SF more readily processed than PLA/SF	DSC used; recycling-related Xc NR	PLA-g-AA/SF had better mechanical properties than PLA/SF due to improved compatibility and ester formation	Water absorption measured; PLA-g-AA/SF showed higher water resistance than PLA/SF	Lower-cost biodegradable composites for packaging, planting cups, disposable products, agricultural films, and mulch films	[51]
PLA-g-MA/green coconut fiber composites based on PLA and recycled natural fibers	Contextual renewable-composite row; not mechanical recycling-cycle study	Biodegradation in *Burkholderia cepacia* compost	GCF dried at 70-80 °C and vacuum dried at 115 °C; PLA-g-MA product dried at 80 °C	No repeated recycling cycles; one blending route	Blend mixing at 180-190 °C for 15 min	PLA-g-MA had slightly lower Mw and intrinsic viscosity than PLA due to grafting	PLA-g-MA/GCF had lower melt viscosity and easier processing	DSC used; recycling-related Xc NR	Compatibilized PLA-g-MA/GCF showed improved compatibility and thermal behavior	Water absorption measured; biodegradation increased with fiber addition	Biodegradable composites using renewable natural fibers	[52]

Ingeo 4032D PLA/PCL blends; optimized 80/20 PLA/PCL composition	Contextual blend-processing study; not recycling or waste study	None	PLA dried at 80 °C for 4 h; PCL dried at 40 °C for 12 h	No recycling cycles	Melt mixing at 180 °C, 60 rpm, 10 min; compression molding at 180 °C followed by fast cooling	NR	Rheology used for blend design; no recycling-related MFI	PLA Xc about 9.7% for neat PLA and 7.2% for PLA/PCL 80/20	PLA/PCL 80/20 gave about 16-fold higher notched impact strength than neat PLA with acceptable stiffness loss	NR	Contextual formulation strategy for improving toughness of PLA recyclates or PLA-based blends	[53]
3D-printing PLA fragments dissolved and plasticized with glycerol; PET fragments hydrolyzed in same study	Contextual chemical/solvent reprocessing study; not mechanical recycling	None	Cast PLA/glycerol films dried at 40 °C overnight	No mechanical recycling cycles; PLA dissolved in chloroform and cast with glycerol	PLA dissolution in chloroform; film drying at 40 °C; PET hydrolysis at 70 °C	NR for PLA molecular weight	NR	NR	Mechanical properties NR	NR	Low-cost valorization of 3D-printing waste by solvent/chemical route; not a core mechanical-recycling row	[54]

Table S2. Chemical and hydrothermal recycling routes

Feedstock	Reaction medium	Catalyst	Temperature and time	Atmosphere	Main product	Yield	Purity	Racemization assessment	Waste/additive tolerance	Repolymerization demonstrated	Scale	Ref.
PLA powder	Hydrothermal water	None	140 °C, 3 h	High-pressure reactor; atmosphere not emphasized	Lactic acid	96% PLA breakdown; 240 g/L LA	HPLC-quantified hydrolysate; isolated purity not reported	Not assessed	Model PLA only	Indirect: hydrolysate used for PHB production by fermentation	Lab high-pressure reactor; shake-flask validation	[57]
PHB powder and PHB-compounded pellets	Hydrothermal water	None	200 °C, 5-6 h	High-pressure reactor	3-hydroxybutyric acid + crotonic acid	72.9-74.0% 3HB and 21.9-27.1% CA in hydrolysates	PHBCP feedstock: 85% PHB purity; product purity not isolated	3HB racemization/decomposition noted as risk at higher temperature/longer time	PHB-compounded pellets tested; lower performance than pure PHB	Yes, hydrolysates converted to PHB; fed-batch reached 8.2 g/L PHB	Lab reactor + 0.5 L working-volume bioreactor	[57]
Virgin PLA and real waste PLA food container	Subcritical water	None	Optimum: N_2, 200 °C, 60 min; air, 250 °C, 30 min	N_2, air; CO2 checked for comparison	Lactic acid; minor carboxylic acids and gases	N_2: 88.96% virgin PLA, 54.58% waste PLA; air: 87.20% virgin, 49.29% waste PLA	HPLC-quantified aqueous products; isolated purity not reported	Stereochemistry highlighted as important but not directly quantified	Real waste PLA tested; lower yield attributed to impurities/additives	Not demonstrated; proposed as secondary raw material for PLA resynthesis	Lab high-pressure/subcritical-water reactor	[58]
PLA fragments, Ingeo 2003D	Hydrothermal water	None	140-180 °C, 10-300 min; effective at 160-180 °C for 120 min	Water-vapour pressure in sealed batch reactor	Water-soluble lactic acid	>95% PLA hydrolyzed to LA within 120 min at 160-180 °C	HPLC showed mainly LA peak; product isolation not reported	Not assessed	Clean model PLA; post-consumer relevance discussed	Not demonstrated	7.7 cm^3 lab batch reactor	[59]
PLA, Ingeo 3052D	Alkaline microwave hydrolysis; 10% w/v NaOH	HTMAB phase-transfer catalyst	100 °C, 10 min optimum; microwave irradiation	N_2-filled PTFE microwave tube	Lactic acid/sodium lactate and oligomers	>90% PLA degradation under optimum conditions	FTIR-confirmed products; isolated monomer purity not reported	Not assessed	Model PLA; not tested on real waste/additives	Not demonstrated	10 mL microwave reactor	[60]

Post-consumer PLA: phone case, toy, film, cup, 3D-printing material	Ethanol + THF	Propylenediamine Zn(II) complex, $Zn(2Pr)_2$	50 or 90 °C; 1-3 h	N_2/inert handling	Ethyl lactate	Highest: 71% from PLA film after 3 h at 50 °C	GC/GPC quantified; isolated purity not reported	Not assessed	Multiple real post-consumer PLA items with unknown additives	Not demonstrated; route to lactide/PLA reformation discussed	Bench stirred reactor; 12.5 g PLA batches	[61]
PLA pellets, NatureWorks Ingeo 6202D	Methanol or ethanol + THF	Zn acetate + DMAP dual catalyst	100-130 °C; time-dependent alcoholysis	N_2-degassed autoclave	Methyl lactate or ethyl lactate	Methanolysis gave alkyl lactate yield >97%; ethanolysis slower/lower under comparable conditions	GC-quantified; product isolation purity not reported	Not assessed	Virgin PLA only; industrial relevance discussed	Not demonstrated; alkyl lactate-to-lactide circular route discussed	300 mL stirred autoclave; 2 g PLA	[62]
PLA pellets, NatureWorks Ingeo 6202D	Methanol + THF	$Zn(OAc)_2$, $Mg(OAc)_2$, TBD, DMAP, and Lewis acid/base catalyst mixtures	90-130 °C; kinetic runs	N_2-degassed autoclave	Methyl lactate	MeLa reached ca. 90% relative concentration at completion; $Zn(OAc)_2$-TBD and $Zn(OAc)_2$-DMAP improved rates	NMR/GC-quantified; isolated purity not reported	Not assessed	Virgin PLA only	Not demonstrated	300 mL stirred autoclave; 2 g PLA	[63]
High-molecular-weight commercial PLA	Bulk molten alcoholysis with pentaerythritol or dipentaerythritol	Zinc stearate	195 °C, 90 min polyol mixing + 60 min after catalyst addition	Argon flow	Hydroxyl-terminated star-shaped PLA oligomers	Mw reduced from 92,000 to about 1,700 g/mol for PE 10 wt% + Zn stearate	Product used without catalyst removal; monomer purity not applicable	Not assessed; starting PLA was 96% L-isomer	Catalyst intentionally retained; formulation without purification tested	Not PLA repolymerization; oligomers reacted with castor-oil triglycidyl ether to make formulations	Lab internal mixer; scalable solvent-free concept	[72]
Biodegradable straw, mostly PLA with PBAT contribution	CO2-assisted thermochemical conversion	None	400-800 °C; 1 min at target temperature	N_2 or CO2	Lactic acid in liquid product; gases, char, wax	LA selectivity up to 14-fold higher in CO2 than N_2; mass yield not given as purified LA	Product selectivity by GC-MS; isolated purity not reported	Not assessed	Mixed commercial straw; PBAT-derived products also observed	Not demonstrated	Lab pyrolysis reactor; 1 g straw	[64]

Biodegradable PLA straw	Thermocatalytic conversion	Sea-shell-derived basic catalyst, mainly $CaCO_3$	Best: 500 °C; catalyst/feedstock mass ratio 0.5	N_2 thermal/catalytic system	Lactic acid	Up to ~12 wt% LA; 130× higher than non-catalytic conversion at optimum	GC-MS quantified; isolated purity not reported	Not assessed	Commercial straw; catalyst from shell waste; coke deactivation after reuse	Not demonstrated	Lab thermocatalytic reactor	[65]
Used biodegradable straws containing PLA/PBAT/PBSA	Catalytic pyrolysis	MSW incinerator bottom ash, CaO-rich	500 °C; 1 min	N_2 or CO2; best in CO2	PLA monomers: lactide + lactic acid	20.84 wt% PLA monomers with MSW-BA in CO2	GC-MS quantified; isolated purity not reported	Not assessed	Used straws and mixed biodegradable polymers; MSW-BA catalyst	Not demonstrated	Continuous-flow/fixed-bed lab pyrolyzer; 1 g feed	[66]
PLA of different molecular weights; PLA cup; reinforced PLA; PHA copolymers	Water	Ru/CeO_2 solid catalyst	PLA: 175 °C, 0.5-1 h; PHA: 200 °C, 0.75 h	5 bar H_2	PLA: lactic acid; PHA: 3-HBA, BA, CA, related products	PLA: 69%, 68%, 55% LA for Mn 8k, 41k, 79k; PLA cup reached 85% LA at 60 min; reinforced PLA 11-57% LA depending additive; PHA products near full conversion at extended time	HPLC/GPC quantified; product isolation purity not reported	Not assessed	Mn, PLA cup, cellulose/nanochitin additives, and PHA copolymers assessed	Not demonstrated; closed-loop monomer recovery proposed	10-50 mL Hastelloy autoclaves	[73]
PLA pellets and PHB powder for microbial-oil bioprocess	PLA: hydrothermal water; PHB: alkaline NaOH	PLA: none; PHB: 0.5 M NaOH	PLA: 140 °C, 4 h; PHB: 80 °C, 30 h	Autoclave/alkaline treatment; atmosphere not emphasized	PLA: lactic acid; PHB: 3HB + CA	PLA hydrolysate ~120 g/L LA, almost complete hydrolysis; PHB ~98% degraded, 63.7% 3HB and 36.3% CA	Hydrolysates filtered; isolated purity not reported	Not assessed	PLA/PHB hydrolysates and blends used as post-consumer-bioplastic model streams	Not polymer resynthesis; hydrolysates converted to microbial oil	Autoclave/bench hydrolysate + shake-flask/fed-batch fermentation	[69]

Water-soluble PLA oligomers, n = 2-9, LL and DL compositions	Acidic aqueous medium, pH 2	Acid-catalyzed hydrolysis by medium	40-120 °C; time-resolved kinetics	Aqueous solution	Lactic acid/short oligomer hydrolysis products	Kinetic constants reported; not a waste-yield study	HPLC quantified oligomer profiles	Yes; chiral composition evaluated; no dependence of hydrolysis constants on chiral composition	Model oligomers only; no additives/waste	Not demonstrated	Analytical/kinetic lab study	[74]
Industrially compostable PLA food-packaging waste, cut into 2 × 2 cm pieces	Aqueous microbial growth medium for hydrolysis, followed by mixed-culture fermentation	No chemical catalyst; mixed microbial culture for fermentation	Hydrolysis: 70 °C, 21 days; fermentation: 30 °C, day 21-58	N_2 flushing; anaerobic fermentation	Lactic acid after hydrolysis; C2-C6 carboxylates after fermentation, mainly n-butyrate	Hydrolysis efficiency: 37.8-39.1%; final lactate: 13.8 g/L from 30 g/L PLA-FPW and 26.6 g/L from 60 g/L PLA-FPW; n-butyrate: 6.5 ± 1.4 g/L from 60 g/L PLA-FPW; carbon recovery into carboxylates up to 92%	HPLC/GC-quantified aqueous products; isolated purity not reported	Not assessed	Real PLA packaging with printed compostability label tested; possible additives did not hinder carboxylate production	Not demonstrated; carboxylates proposed as substrates/building blocks for PHA or other products	Serum-flask laboratory proof-of-concept	[70]
Commercial PLA pellets/products; mixed PLA with PET, PP, and PE; other polyesters/polycarbonates	Small amount of water; solvent-free hydrolysis	Diphenyl phosphate, DPP, 3.5 wt%	PLA-to-oligomer route: 160 °C, 1.5 h; further hydrolysis to lactic acid with additional water and prolonged reaction	No inert gas or external pressure required	Oligo(lactic acid), OLA; lactic acid solution; lactide/PLA via closed-loop route	OLA DP <3; conversion >85%; 10 reuse cycles with final conversion 95.6%; kg-scale conversion >90%; lactic acid solution comparable to commercial quality	Lactic acid solution comparable to commercial product after washing; repolymerized PLA showed high Mw and narrower dispersity than original PLA	Yes; optical purity/configuration retention assessed by specific rotation; no racemization under reported conditions	Commercial PLA products and mixed plastics tested; PET/PP/PE did not compromise PLA hydrolysis and were separated by filtration	Yes; OLA directly converted to lactide and repolymerized into PLA with reported high molecular weight	Gram-scale and kilogram-scale; 5 L reactor	[67]

Commercial PLA pellets/products, PLA trimmings, and mixed PLA with durable plastics; also PBAT, PBS, PCL, PTMC, PBSA, PHB	Small amount of water; solvent-free hydrolysis	p-bis-nitrophenyl phosphate, p-BNPP, 3.5 wt%	PLA hydrolysis: 160 °C, 30 min; further OLA hydrolysis: 95 °C, 9 h	No external pressure or organic solvent; no gas protection required	OLA; concentrated lactic acid solution; lactide/PLA via closed-loop route	DP <4, >80% conversion, 10 reuse cycles; kg-scale OLA; secondary hydrolysis: ~79 mol% LA	Concentrated lactic acid solution; repolymerized PLA obtained with high molecular weight; product quality assessed by NMR, HPLC, GPC, DSC	Yes; optical purity retained, with L-configuration ratio close to original PLA; no significant racemization reported	PLA cups, 3D-printing materials, yarn, masks, shopping bags, and nonwoven fabric tested; durable plastics separated by filtration	Yes; OLA converted to lactide and then repolymerized to PLA	Gram-scale and kilogram-scale; 5 L batch reactor	[68]

Table S3. Environmental and, where available, economic assessments of PLA/bioplastic recycling and end-of-life options. Abbreviations: MR, mechanical recycling; CR, chemical recycling/depolymerization; MSWI, municipal solid-waste incineration; AD, anaerobic digestion; FU, functional unit; GWP, global warming potential; EoL, end of life; NR, not reported; N/A, not applicable.

**Ref.**	**System boundary**	**Functional unit**	**Geographical setting**	**Energy mix**	**Recycling route**	**Virgin-material substitution ratio**	**Climate-change result**	**Other impact categories**	**Sensitivity assumptions**	**Identified burden shifting**
[75]	Attributional EoL LCA from PLA waste source through collection, sorting, transport, recycling or MSWI; recyclate/energy credits included.	1 t PLA waste treated.	Germany.	German energy and power mix; European datasets where German data unavailable.	PIW-MR, PCW-MR, solvent-based recycling, CR; MSWI reference.	Quality-corrected virgin PLA substitution; route-specific correction factors used for recyclate quality.	In the modeled cases, PLA recycling routes showed lower GWP than MSWI; GHG savings from virgin PLA replacement were 0.3-1.2 times higher than incineration savings.	Primary energy demand, fossil resource depletion, agricultural land occupation, photochemical ozone formation, acidification, eutrophication, particulate matter.	PLA share in lightweight packaging, sorting quota, recyclate quality, correction factor, energy recovery, scale-up of CR and solvent recycling.	Benefits depend on clean PLA streams and substitution credits; burdens shift to sorting, washing, drying, purification, and process energy.
[76]	Harmonized/remodeled LCA comparison of published PLA depolymerization studies; EoL stages grouped as collection, pretreatment, depolymerization.	1 Mg PLA waste treated.	Harmonized to Germany/Europe; original studies varied.	Harmonized ecoinvent v3.9.1; German/European electricity and heat assumptions tested.	Hydrolysis, alcoholysis, lactide/oligomer depolymerization routes.	Substitution products varied: lactic acid, PLA, or DMF; quality/substitution factors study-specific.	Harmonized GWP with credits ranged from −2869 to −1378 kg CO2-eq/Mg PLA waste.	Mainly GWP; other categories limited by inconsistent original inventories.	LCI database, LCIA method, software, geographical scope, energy mix, substitution product, catalyst/additive data.	Results are credit- and database-dependent; omitted additives/catalysts and generic datasets can shift or mask burdens.

[78]	Cradle-to-grave carbon-footprint model including bio-carbon uptake, production, transport, and EoL.	1 kg bio-based plastic.	Shanghai/China.	China 2020 electricity baseline plus decarbonization scenarios.	Incineration with energy recovery, MR, CR, composting, AD.	MR/CR credited through avoided products/material recovery; explicit quality ratio NR.	For PLA, incineration was better than CR under carbon-intensive electricity; CR became superior under renewable-rich electricity.	GWP100a and CO2 uptake carbon accounting.	Electricity decarbonization, diesel/electric transport, degradation kinetics, carbon accounting, CCS and EoL technology assumptions.	Route preference changes with energy mix: incineration loses stored biogenic carbon, while CR is energy-intensive unless power is decarbonized.
[87]	Cradle-to-grave LCA plus eco-efficiency/cost for PLA, PHA, PBS, and PP food boxes.	1000 takeaway food boxes with lids, 650 mL.	Thailand; sugarcane/cassava feedstocks.	Thai LCI and ecoinvent data.	Current EoL, 100% composting, 100% MR, 100% incineration/ WTE scenarios.	100% MR modeled as reducing virgin resin demand; quality ratio NR.	In the modeled 100% scenario, MR gave the lowest environmental and eco-efficiency burdens; composting was less favorable than MR.	18 ReCiPe midpoint categories and 3 endpoint categories; product cost.	Resin cost, feedstock allocation, EoL route, electricity cost, recycling infrastructure.	Climate/fossil-resource benefits can shift to land occupation, eutrophication, toxicity, and cost burdens from agricultural feedstocks.
[88]	Attributional cradle-to-grave LCA of biopolymers and fossil plastics; production plus EoL; product formation/use excluded.	1 kg polymer.	United States; Phoenix-area EoL case.	US production and EoL datasets.	PLA/TPS composting or landfill; PET/PE recycling or landfill.	PET/PE recycling credited by avoided virgin resin; PLA recycling not modeled due lack of infrastructure.	Recycling gave substantial GWP and fossil-fuel benefits in the modeled comparison; PLA landfill methane potential	Eutrophication, water use, smog, ozone depletion, acidification, carcinogens/non-carcinogens, respiratory effects, ecotoxicity, fossil fuel depletion.	Travel distance, sorting technology, landfill degradation, methane capture, recycling allocation.	Replacing recyclable plastics with compostable PLA can lose recycling credits; composting may increase transport and

							affected climate results.			diesel-related burdens.
[89]	Cradle-to-grave LCA of PLA and PET drinking-water bottles; polymer production, transport, bottle forming, collection/sorting/EoL.	1000 bottles of 500 mL.	Italy/Europe with NatureWorks PLA data.	SimaPro/Ecoindicator 99; non-renewable electricity assumption for PLA.	MSWI, MR, landfill for PLA/PET; composting for PLA.	PLA recycling scenario assumed high virgin PLA displacement; PET recycling credited by avoided production.	PLA advantage mainly came from renewable feedstock/fossil-resource reduction; benefit depended on high recycling.	Human health, ecosystem quality, resources; climate change, ecotoxicity, acidification/eutrophication, land use, minerals, fossil fuels.	Disposal shares, degradation in landfill/composting, biogas recovery, weighting and inventory uncertainty.	PLA shifts burdens to pesticides, fertilizer, land and water use; PLA/PET sorting remains critical.
[90]	Cradle-to-grave LCA of PLA, PP, PET single-use cups and reusable stainless-steel cup.	One 650 mL drink container per workday for 1 year; 260 uses.	Thailand.	Thai electricity; renewable/recycled-content scenarios.	Recycling, incineration/RDF, composting/AD where relevant; stainless-steel reuse and recycling.	Cut-off approach; recycled-content scenarios included 25% recycled plastic and 50% recycled stainless steel.	PLA cup had lower GWP/fossil depletion than PP/PET; stainless steel became better after sufficient reuse; 25% recycled content reduced impacts by 35-56%.	GWP, fossil depletion, human toxicity, terrestrial acidification.	Number of reuses, washing mode, recycled content, consumer behavior.	Reuse shifts burden to washing energy/water; PLA benefits depend on proper EoL and can be lost under methane-producing mismanagement.

[91]	Cradle-to-grave LCA of disposable and reusable tableware; raw materials and EoL included; converting and transport excluded.	Service of 1000 meals.	Italy/Europe.	Italian electricity for washing; renewable electricity discussed.	No recycling due food residues; PLA-PBS composting; PP/PS landfill; reusable PLA-PBS washed/reused.	N/A; no recycling modeled.	Compostable sets were generally lower than fossil sets except ozone depletion and aquatic eutrophication; reusable performance depended on reuse and washing.	GWP100a, ozone depletion, ozone formation, acidification, aquatic eutrophication, human toxicity water, chronic ecotoxicity water.	Number of reuses, washing mode, renewable power for production/washing.	Disposable systems shift burdens to production/EoL; reusable systems shift burdens to washing; compostables can shift burdens to eutrophication/ozone categories.
[92]	Cradle-to-grave LCA plus TEA for PLA composites with organic and inorganic fillers.	Per kg/part; 100,000 kg lot; part sizes 0.01, 0.1, 1 kg.	United States model/database context.	Plastic Comparator + EIO-LCA; US industry energy/cost datasets.	Recycling, incineration, landfill, landfill + methane recovery, no EoL.	Recycling factor used; explicit virgin-resin substitution ratio NR.	Organic fillers with recycling, especially DDGS/wood, gave the lowest modeled environmental and economic burdens; glass plus landfill gave the highest burdens in that assessment.	GWP, air acidification, air/water eutrophication, ozone depletion, smog, carcinogens, non-carcinogens, energy intensity, cost.	Filler type/price, part size, processing cost, EoL treatment.	Inorganic fillers may improve properties but increase cost/environmental intensity; bio-fillers reduce impacts but introduce drying/processing and property trade-offs.

[96]	System-level linear-programming model for plastics in Japan; raw material extraction to 12 EoL options.	Annual plastic demand by application; kg-plastic basis in optimization.	Japan.	Japanese inventory/EoL system; decarbonized EoL scenarios.	MR, feedstock recycling, blast furnace/coke oven, pyrolysis, gasification, RPF, cement fuel, incineration, open dumping/biodegradation.	Depends on route: virgin polymer, coal/coke, oil, ammonia, or heat displaced.	Optimized bio-based plastic strategies reduced life-cycle GHG; unconstrained material-property case gave −15.5 million t CO2-eq/year.	Climate/LCGHG only.	Material-property constraints, product application, recycling capacity, product mass, EoL decarbonization.	Climate-only optimization may favor feedstock recycling where fossil coal/coke displacement is high, but may ignore land, water, toxicity, or cost trade-offs.
[95]	Supporting environmental-hazard study; non-LCA chemical/ecotoxicity screening of conventional, recycled and alternative plastics.	N/A; leachate-based testing.	Spain/Galicia laboratory-market context.	N/A.	N/A.	N/A.	N/A.	Sea-urchin embryo toxicity, GC-MS chemical profile, chemical hazard indices.	Polymer/additive composition and leachate preparation.	Compostable or “green” alternatives may shift burden from persistence/climate to chemical hazard and aquatic ecotoxicity.
[82]	Supporting sorting study; NIR classification of PLA and conventional lightweight packaging plastics.	N/A; lab-scale sorting/classification.	Germany.	N/A.	NIR sorting of PLA from PP, HDPE, PET, PS.	N/A.	N/A.	Pixel-based classification accuracy, sortability of degraded and non-degraded PLA.	Degradation level, LWP composition, spectral classification.	Recycling benefits require reliable sorting; degraded PLA needs to remain identifiable to avoid contamination and recyclate quality losses.

[83]	Supporting a recyclate-quality study; simulated PLA contamination in mixed polyethylene recycling.	N/A; MPE/PLA blends.	Hungary.	N/A.	Mechanical reprocessing of MPE contaminated with PLA.	N/A.	N/A.	Rheology, viscosity, specific volume, tensile strength, modulus, elongation, impact strength.	PLA contamination level, processing conditions.	PLA contamination lowers elongation/impact strength and changes viscosity, creating quality-loss burden shifting in conventional recycling streams.
[84]	Supporting recyclate-quality study; TPS/PLA contamination in HDPE, PP, PET recycling.	N/A; lab-scale blends.	Sweden.	N/A.	Mechanical recycling simulation of conventional plastics contaminated with TPS/PLA.	N/A.	N/A.	Tensile, Charpy impact, DSC, SEM.	1-5 wt% contamination, humidity conditioning, polymer type.	PET was sensitive to contamination; even low bioplastic contamination can downgrade recyclate quality.
[77]	Prospective EoL LCA; begins when post-consumer PLA waste reaches treatment facility; includes sorting, shredding, washing/drying, treatment, residual waste; excludes upstream PLA	1 kg post-consumer PLA waste treated.	Europe-oriented prospective modeling, 2025-2050.	SSP1, SSP2, SSP2-RCP1.9 prospective background systems; decarbonization and learning considered.	Industrial composting, incineration, MR, CR with repolymerization, CR without repolymerization.	Quality-adjusted credits; MR uses reduced substitution for recycled PLA; CR/CRRP credited according to recovered lactic acid or regenerated PLA.	MR gave the lowest modeled climate impact, -1.22 to -0.67 kg CO_2-eq/kg PLA; CR without repolymerization gave the highest modeled climate impact, up to 0.58 kg CO_2-eq/kg PLA.	Multi-impact pLCA including climate, toxicity/ecotoxicity, resource and water-related categories.	SSP pathway, normative learning rate, decarbonization, PLA market penetration, substitution credit, quality correction.	Recycling lowers climate impacts but can raise water use up to 49%; composting showed terrestrial ecotoxicity burden shifting of 31%; net-zero needs high learning rates for most routes.

	production and use.								
[81]	Cradle-to-grave A-LCA and C-LCA of 2G PLA from wheat straw; includes cultivation, straw pretreatment, lactic acid/PLA production and chemical recycling; excludes product manufacturing/ use.	1 kg PLA.	Apulia, Italy wheat straw; European biorefinery context.	Ecoinvent v3.8/SimaPro; cogeneration steam included.	Chemical recycling of PLA waste to lactic acid: sorting, washing, drying, grinding, hydrolysis, centrifugation, evaporation.	Recycled lactic acid offsets virgin lactic acid demand; base case recycling plant supplies 80% of LA demand.	Recycling integration reduced GWP from 1.38 to 0.44 kg CO2-eq/kg PLA.	ReCiPe midpoint/endpoint categories including toxicity, eutrophication, acidification, land use, fossil scarcity, water consumption, human health, ecosystem quality, resources.	Straw cellulose content, straw price/allocation, PLA-waste transport distance, recycling level, Monte Carlo uncertainty, C-LCA substitution scenarios.	Recycling reduces attributed impacts, but straw diversion can shift burdens to animal feed, fertilizer, heat production, and iLUC emissions.
[79]	Cradle-to-grave LCA of flexible sanitary-product packaging; raw materials, supplier transport, extrusion/printing/welding, shipping materials and EoL; excludes consumer distribution/use.	One flexible package containing ~10 sanitary pads.	Italy; primary data from northern Italian packaging company.	SimaPro 9.4, Ecoinvent 3.7; company electricity and natural gas.	Internal post-industrial LDPE recycling; LDPE/R-LDPE recycling, energy recovery, landfill; bioplastic composting/landfill.	R-LDPE contains 40% post-industrial recycled LDPE; explicit substitution ratio NR.	R-LDPE had the lowest overall impacts in the modeled comparison; raw materials and packaging production dominated impacts.	GWP, water consumption, acidification, abiotic depletion of elements and fossil fuels.	Real Italian EoL vs ideal EoL; RecyClass recyclability; composting/recycling rates; supplier distance; process scrap.	Compostable bioplastics did not automatically outperform plastics; burdens shift to raw-material production and incomplete composting/landfill.

[80]	Cradle-to-grave LCA of PET, PLA and refillable aluminium water bottles; raw material production, forming, transport, use/washing and EoL.	One year of drinking-water use: 1095 PET or PLA bottles, or 0.4 aluminium bottle assuming 2.5-year lifetime.	Italy.	OpenLCA/ ReCiPe midpoint; Ecoinvent; hot-water washing scenarios.	PET open-loop/closed-loop recycling, incineration, landfill; PLA composting/incineration; aluminium reuse.	PET recycling credited by avoided virgin material; PLA composting no material substitution credit.	PET had lower modeled impacts than PLA and aluminum when daily washing was included; PET recycling reduced GWP by up to ~30%, while PLA composting gave no GWP saving.	Ten categories including GWP, eutrophication, human toxicity and ecotoxicity.	Aluminium lifetime, washing with hot water vs soap, PET recycling route, PLA composting vs incineration.	PLA shifts burden to corn/agriculture; aluminium reuse shifts burden to washing energy and hygiene; PET benefits depend on collection/recycling.
[85]	Supporting sorting study; lab-scale NIR identification and sorting of PLA.	N/A; virgin PLA, conventional plastics and 10 PLA product samples.	Austria; Montanuniversität Leoben.	N/A.	NIR sorting of PLA from HDPE, PET, LDPE, LLDPE, TPU, PP, PVC.	N/A.	N/A.	Spectral separation, detection/ejection performance, grade/thickness effects.	PLA grade, thickness, fillers, transparency, backlight intensity.	Transparent PLA required correct backlight settings; sorting infrastructure is a prerequisite for realizing LCA recycling benefits.

[86]	Supporting contamination study; lab-scale mechanical recycling simulation of HDPE with PLA contamination and UVA aging.	N/A; HDPE sheets with 0-10 wt% PLA.	Australia.	N/A.	Mechanical recycling of HDPE contaminated with PLA.	N/A.	N/A.	Tensile strength, Young's modulus, toughness, FTIR, wettability, color, surface morphology.	PLA contamination level and 28-day UVA exposure.	PLA contamination can downgrade HDPE recyclate: 10% PLA reduced tensile strength by ~50%; after UVA, 2.5% PLA caused ~51% reduction.
